# Development and evaluation of nanobody tracers for noninvasive nuclear imaging of the immune-checkpoint TIGIT

**DOI:** 10.3389/fimmu.2023.1268900

**Published:** 2023-09-20

**Authors:** Katty Zeven, Timo W.M. De Groof, Hannelore Ceuppens, Robin Maximilian Awad, Thomas Ertveldt, Wout de Mey, Fien Meeus, Geert Raes, Karine Breckpot, Nick Devoogdt

**Affiliations:** ^1^ Laboratory of Molecular Imaging and Therapy (MITH), Vrije Universiteit Brussel (VUB), Brussels, Belgium; ^2^ Laboratory for Molecular and Cellular Therapy (LMCT), Department of Biomedical Sciences, Vrije Universiteit Brussel (VUB), Brussels, Belgium; ^3^ Laboratory for Cellular and Molecular Immunology (CMIM), Vrije Universiteit Brussel (VUB), Brussels, Belgium; ^4^ Myeloid Cell Immunology Lab, Vlaams Instituut voor Biotechnologie (VIB) Center for Inflammation Research, Brussels, Belgium

**Keywords:** TIGIT, immune checkpoint (ICP), nuclear imaging, noninvasive diagnosis, tracer development, nanobodies

## Abstract

**Introduction:**

T cell Ig and ITIM domain receptor (TIGIT) is a next-generation immune checkpoint predominantly expressed on activated T cells and NK cells, exhibiting an unfavorable prognostic association with various malignancies. Despite the emergence of multiple TIGIT-blocking agents entering clinical trials, only a fraction of patients responded positively to anti-TIGIT therapy. Consequently, an urgent demand arises for noninvasive techniques to quantify and monitor TIGIT expression, facilitating patient stratification and enhancing therapeutic outcomes. Small antigen binding moieties such as nanobodies, are promising candidates for such tracer development.

**Methods:**

We generated a panel of anti-human or anti-mouse TIGIT nanobodies from immunized llamas. In addition, we designed a single-chain variable fragment derived from the clinically tested monoclonal antibody Vibostolimab targeting TIGIT, and assessed its performance alongside the nanobodies. *In vitro* characterization studies were performed, including binding ability and affinity to cell expressed or recombinant TIGIT. After Technetium-99m labeling, the nanobodies and the single-chain variable fragment were evaluated *in vivo* for their ability to detect TIGIT expression using SPECT/CT imaging, followed by ex vivo biodistribution analysis.

**Results:**

Nine nanobodies were selected for binding to recombinant and cell expressed TIGIT with low sub-nanomolar affinities and are thermostable. A six-fold higher uptake in TIGIT-overexpressing tumor was demonstrated one hour post- injection with Technetium-99m labeled nanobodies compared to an irrelevant control nanobody. Though the single-chain variable fragment exhibited superior binding to TIGIT-expressing peripheral blood mononuclear cells *in vitro*, its *in vivo* behavior yielded lower tumor-to-background ratios at one hour post- injection, indicating that nanobodies are better suited for *in vivo* imaging than the single-chain variable fragment. Despite the good affinity, high specificity and on-target uptake in mice in this setting, imaging of TIGIT expression on tumor- infiltrating lymphocytes within MC38 tumors remained elusive. This is likely due to the low expression levels of TIGIT in this model.

**Discussion:**

The excellent affinity, high specificity and rapid on-target uptake in mice bearing TIGIT- overexpressing tumors showed the promising diagnostic potential of nanobodies to noninvasively image high TIGIT expression within the tumor. These findings hold promise for clinical translation to aid patient selection and improve therapy response.

## Introduction

Besides classical cancer treatment, immunotherapy has gained significant attention in recent decades. Immune checkpoint (ICP) blockade therapies (ICBs) using monoclonal antibodies (mAbs) against programmed cell death-1 (PD-1) and its ligand (PD-L1), lymphocyte activating gene-3 (LAG-3) or cytotoxic T-lymphocyte-associated protein-4 (CTLA-4) have demonstrated remarkable clinical impact. Certain ICBs have even emerged as a first-line treatment for patients with metastatic melanoma, non-small cell lung carcinoma (NSCLC), and renal cell carcinoma (RCC) as monotherapy or combination therapy (1–3). ICPs play a crucial role in maintaining immune homeostasis by initiating activating or inhibitory signals downstream upon interaction with their ligands. Within the dynamic tumor environment, these inhibitory ICPs are expressed to inhibit cytotoxic T cells and evade immune cell attacks. By disrupting the interactions of ICP receptors and their ligands, the brake on the immune cells can be released, and T-cell dysfunction can be reversed (3–5). Despite the success of currently available ICBs, some patients fail to respond due to primary and/or acquired resistance (6). Expression of different ICPs on metastatic sites or upregulation of other ICPs following ICB is often noticed (7, 8). Consequently, other ICPs are being explored as potential therapeutic targets to enlarge the treatment possibilities (9, 10).

T cell immunoglobulin (Ig) and ITIM domain receptor (TIGIT), also known as VSIG9, Vstm3, and WUCAM, is a next-generation inhibitory ICP from the immunoglobulin superfamily (11). TIGIT is mainly expressed on T cell subsets, including regulatory T cells (Tregs) (12), activated CD8^+^ and CD4^+^ T cells, and natural killer (NK) cells (13, 14). Additionally, its expression has been observed on B cells (15, 16), memory T cells, and follicular T helper cells (17, 18). Upregulated TIGIT expression has been associated with poor prognosis of multiple cancers such as gastric cancer, melanoma and hepatocellular carcinoma (HCC) (19, 20). TIGIT interacts with multiple ligands, including CD112 (21) and CD113 (22). However, the principal ligand for TIGIT is CD155 (14), which is also the ligand of the activating receptor CD226. TIGIT interacts with CD155 with at least 100 times higher affinity (K_D_ = 1-3 nM) compared to the co-stimulatory receptor CD226 (K_D_ = 119 nM). Consequently, TIGIT-CD155 interaction initiates downstream inhibitory signaling, limits T-cell response and downregulates NK-cell function (14, 23).

Currently, multiple monoclonal antibodies (mAb) have been developed to interrupt the interactions between TIGIT and its ligands (24). Following positive preclinical results, numerous clinical trials are ongoing to investigate TIGIT blockade in patients with solid or hematological cancers, either as monotherapy or combined with another ICB. In patients with NSCLC, anti-TIGIT mAb Tiragolumab (Genentech) in combination with anti-PD-L1 mAbs Atezolizumab (Roche) demonstrated prolonged survival in a phase II clinical trial (25). The anti-TIGIT mAb Vibostolimab (Merck) was evaluated in patients with NSCLC as monotherapy or in combination with the anti-PD-1 mAb pembrolizumab (Merck) in a phase I study (NCT02964013), showing objective response rate of 26% when combined with pembrolizumab (26). Other clinical trials also supported the promising clinical benefit of combining TIGIT blockade with PD-1/PD-L1 blockade (27). The latest development showed that Tiragolumab combined with Atezolizumab and the anti-VEGF mAb Bevacizumab (Roche) significantly increased the overall response of patients with unresectable HCC compared to the control arm (NCT04524871) (28). These results indicate the potential of anti-TIGIT mAbs as novel ICB (26, 27).

Immunohistochemistry (IHC) analysis of patient biopsies is commonly utilized to evaluate the expression of ICPs as predictive markers for therapy response. However, IHC is not always representative for the dynamic ICP expression pattern, and the biopsy required for IHC is invasive for the patient. Moreover, the primary tumor lesion is not always easily accessible, and the metastatic sites can display distinct expression profiles. Hence, there is a need for a reliable method to detect ICPs that circumvent the shortcomings of IHC. Niemeijer et al. showed that evaluating PD-L1 expression in patients with NSCLC using radiolabeled mAbs with nuclear imaging could predict the response rates of anti-PD-1/L1 therapies. However, the extended time required for imaging with mAbs (160 hours post-injection) raises the need for a more time-efficient and lower radiation burden approach (29). Small antigen binding fragments, such as Nanobodies (Nbs) derived from the heavy-chain only antibodies of *Camelidae*, are promising alternatives. Nbs have a molecular weight of only 10-15kD which is far below the glomerular filtration rate resulting in a fast pharmacokinetic profile (30). Nbs have been employed for diagnostic purposes, showing high contrast imaging within a short time after tracer injection. Multiple Nbs have already entered clinical evaluation, such as Nbs against the breast oncoprotein human epidermal growth factor receptor 2 and the macrophage marker CD206 as PET imaging probes (31–33). Previously, we preclinically evaluated Nb radiotracers against the ICPs PD-L1 and LAG-3, demonstrating specific tumor uptake and high contrast imaging of ICPs on cancer or immune cells in syngeneic tumor models (34–37). The development of these Nbs into PET tracers has further highlighted their potential as specific imaging agents for ICPs (38).

The observation that only a fraction of patients exhibits a favorable response to anti-TIGIT mAbs in clinical trials underscores the need for methods to track TIGIT expression and follow-up patient response. This can be performed noninvasively by nuclear imaging. In this study, we described the generation of anti-TIGIT Nbs which we characterized for their diagnostic potential upon radiolabeling with Technetium-99m (^99m^Tc). The binding affinities and stabilities of the Nbs were evaluated. Moreover, we also evaluated the *in vivo* specificity of the Nbs in human TIGIT knock-in mice and the ability to image TIGIT in tumor bearing mice.

## Materials and methods

### Cell lines

The human embryonal kidney (HEK) 293T cell line and the mouse colorectal carcinoma (CRC) MC38 cell line were obtained from the American Type Culture Collection (ATCC, Molsheim Cedex, France) and cultured in Dulbecco’s modified Eagle’s medium (DMEM) supplemented with 10% FBS (Harlan, Horst, The Netherlands), 2 mmol/L L-Glutamine (Sigma-Aldrich, Zwijndrecht, Belgium), 100 U/mL penicillin, 100 μg/L streptomycin (PS; Sigma-Aldrich, Zwijndrecht, Belgium).

The mouse lung carcinoma cell line TC-1 was provided by T.C. Wu (John Hopkins University, Baltimore, MD, USA) and cultured in RPMI 1640 medium supplemented with 10% FCI (Harlan, Horst, The Netherlands), 2 mmol/L L-Glutamine (L-Glu; Sigma-Aldrich, Zwijndrecht, Belgium), 100 U/mL penicillin and 100 μg/ml streptomycin (PS; Sigma-Aldrich, Zwijndrecht, Belgium), 1 mmol/L sodium pyruvate and non-essential amino acids (Sigma-Aldrich, Zwijndrecht, Belgium), 1 mM G418 (Thermofisher Scientific, Asse, Belgium), 5 mM HEPES (Thermofisher Scientific) and 50 μM beta-mercaptoethanol (Thermofisher Scientific).

The cells were cultured at 37°C 5% CO_2_.

### Mice

6-12 weeks old C57BL/6J mice and Swiss nude Crl : Nu(Ico) Foxn1nu mice were purchased from Charles River (Ecully, France). Breeding pairs of C57BL/6-*TIGIT* ^em1(hTIGIT)Smoc^ (Cat. NO. NM-HU-00053) were purchased from Shanghai Model Organisms Center, Inc. (Pudong, Shanghai, China). These mice were referred to as hTIGIT knock-in (KI) mice. All projects using mice were approved by the Ethical Committee for Use of Laboratory Animals of the VUB (file references: 20-272-10; 22-272-7).

### Lentiviral vector production and transduction

Transfer plasmids pHR’ encoding mouse TIGIT (mTIGIT) or human TIGIT (hTIGIT) were generated using the Gibson assembly method based on designed gBlocks from Integrated DNA Technologies (IDT) to contain the coding sequence for mTIGIT (NM_001146325) or hTIGIT (NM_173799) flanked by 20 base pairs overhangs. The packaging plasmid pCMVΔR8.9 and the VSV.G encoding plasmid pMD.G were a gift from D. Trono (University of Geneva, Switzerland). Lentiviral vectors were produced following transfection of HEK293T cells. Following their characterization, lentiviral vectors were used to transduce HEK239T cells and TC-1 cells as described previously (39).

### Generation and isolation of TIGIT specific Nbs

Nbs were generated and isolated as previously described (40). Briefly, immunization and selection of the Nbs were performed in collaboration with the VIB Nanobody Core (Brussels, Belgium). Two llamas were subcutaneously immunized six times at a weekly interval with 100 µg of recombinant mouse (Biolegend, Cat. No. 771808) and human (Biolegend, Cat. No. 768608) TIGIT-Fc carrier-free proteins, mixed with Gerbu adjuvant. Blood was collected on day 40 for lymphocyte preparation and generation of the Nb libraries to screen for TIGIT-specific binders. To create the Nb libraries, total RNA from blood lymphocytes was used as a template for cDNA synthesis with an oligo(dT) primer using PCR. The amplicons were cloned into the phagemid vector pMECS. Once the libraries were generated, Nbs were displayed on M13 bacteriophages and three rounds of panning were performed on biotinylated m/hTIGIT-Fc proteins. To avoid enrichment of the Fc region phages, non-biotinylated human IgG1 Fc was added (Sino Biological, Cat. NO. 10702-HNAH). Crude periplasmic extracts including soluble Nbs were produced and evaluated for binding to m/hTIGIT-Fc in ELISA.

### Design and production of the single chain variable fragment

Sequences of the variable domain of the heavy chain (V_H_) and light chain (V_L_) of the clinically tested mAb Vibostolimab (Merck) were obtained from patent (WO2016028656A1) (41) and ordered as gBlocks from Integrated DNA Technologies (IDT). The V_H_ and V_L_ were linked using a (G4S)_3_ linker and cloned into the pHen6 vector with a C-terminal hexahistadine (HIS_6_)-tag using the Gibson assembly method. The scFv was produced side-by-side with the Nbs.

### Production and purification of the Nbs

The production and purification of the Nbs were described previously (42). The sequences of the nine selected Nbs can be found in **Supplementary Figure 1**. Briefly, the sequences of the selected Nbs were PCR amplified using primers A6E (5′ GAT GTG CAG CTG CAG GAG TCT GGG GGA GG 3′) and PMCF (5′ CTA GTG CGG CCG CTG AGG AGA CGG TGA CCT GGG T 3′) using PCR. cDNA of the selected Nbs were cloned into pHen6 plasmid to encode a C-terminal HIS_6_-tag and transformed into WK6 *E. coli* for large-scale production in one liter of the terrific broth medium. Periplasmic extracts containing soluble Nbs were generated by osmotic shock with Tris-EDTA-Sucrose solution. The Nbs were affinity-purified on HisPur Ni-NTA resin (Thermofisher Scientific, Asse, Belgium) and eluted with imidazole. The suspension was loaded on a size exclusion chromatography (SEC) column (Superdex 75 10/300GL column, Cytiva, Marlborough, MA, USA) and purified using the AKTA high performance liquid chromatographer (GE Healthcare) in PBS. Purity of the Nbs was evaluated using 16% sodium dodecyl sulfate polyacrylamide gel electrophoresis (SDS-PAGE) under reducing conditions, followed by staining using InstantBlue Coomassie protein stain (Abcam, Cambridge, UK). The scFv and an irrelevant control Nb R3B23 (43) were produced in the same way.

### Inoculation of TC-1 and MC38 tumors

7x10^5 unmodified TC-1 and mTIGIT or hTIGIT overexpressing TC-1 cells were injected subcutaneously in Swiss nude Crl : NU(Ico) Foxn/nu mice under isoflurane anesthesia. Similarly, 1x10^6 MC38 cells were inoculated subcutaneously in C57BL/6 mice. Tumor growth was measured using an electronic caliper and tumor volume was calculated using the formula (length x width^2^)/2.

### Flow cytometry

Staining of cell surface markers was performed as previously described (39). A list of the antibodies used to perform stainings is provided in **Supplementary Table 2**. Live/dead staining of the tumor, spleen and lymph node single cell suspensions was performed using the Viobility fixable dye 405/520 (Miltenyi Biotec B.V., Leiden, The Netherlands), REAfinity recombinant antibodies were used for surface stainings (Miltenyi Biotec B.V.).

For Nb binding studies, 900 nM of the purified Nbs in PBS were incubated for one hour at 4°C with mouse or human CD3/CD28 dynabeads (ThermoFisher Scientific) activated splenocytes or human peripheral blood mononuclear cells (PBMCs) respectively. LIVE/DEAD™ Fixable Violet dead cell stain kit (Thermofisher Scientific, L34955) or the Viobility fixable dye 405/520 (Miltenyi Biotec B.V.) was used for live/dead staining of the PBMCs or splenocytes, respectively. Human FcR blocking reagent (Miltenyi Biotec B.V. 130-059-901) or purified anti-mosue CD16/32 (Biolegend, 101301) FcR block was used prior to surface stainings of the PBMCs or splenocytes, respectively. PE-labelled anti-His antibody (Miltenyi Biotec B.V.) was used to detect Nb binding. APC-labeled anti-HA antibody (Biolegend) was used to detect Nb binding during the first screenings.

The BD Celesta flow cytometer (BD Biosciences) was used to analyze samples. Data analysis was performed with FlowJo (Tree Star, Inc, Ashland, OR, USA).

### Single cells of tumor, spleen and lymph nodes

Preparation of single cell suspensions of tumors, spleens and lymph nodes for flow cytometry analysis according to the protocols of Miltenyi Biotec. Briefly, tumors were cut into pieces of approximately 5 mm and transferred to gentleMACS C tubes containing 5 ml ice-cold RPMI1640 supplemented 100 µl collagenase I and 1000 U/mL DNAse I. Tumors were homogenized on the gentleMACS™ OCTO-dissociator for 45 minutes at 37°C. Cells were filtered through a 70 µm filter (BD Falcon) in PBS (Sigma-Aldrich) and centrifuged for 5 minutes at 1500 rpm. Red blood cells were eliminated using the ACK buffer (0.16 M NH_4_Cl, 0.17 M Tris, pH 7.2). Lymph nodes and spleens were minced through a 70 µm filter (BD Falcon) in 5 mL PBS containing 1000 U/mL DNAse I (StemCell), red blood cells were eliminated using the ACK buffer. Cells were kept in ice-cold PBS for further analysis.

### Surface plasmon resonance

The dynamic affinity of the anti-TIGIT Nbs was determined using a Biacore T200 device (GE Healthcare, Machelen, Belgium). The running buffer was HEPES buffered saline at pH 7.4 (HBS, 0.01 M HEPES, 0.15 M NaCl, 3 mM EDTA, 0.005% Tween 20). 10 µg/mL of recombinant m/hTIGIT-Fc proteins (Biolegend) in 10mM Na-acetate pH 4.0 were immobilized on a CM5 sensor chip (Cytiva). Eight times two-folds serial dilutions from 200 nM of the Nbs were injected and analyzed with a flow rate of 10 µl/min at 25°C. The chip was regenerated with two cycles of 0.1 M Glycine HCl pH 2.5 buffer for 15 sec each with flow rate of 30 µl/min and stability time of 30 sec. The mathematical fitting model 1:1 binding with drift and RI2 was used to determine the equilibrium dissociation constant (K_D_) using the BIACORE evaluation software (Cytiva).

### Technetium-99m labeling of the nanobodies

Labeling of the Nbs with ^99m^Tc was performed with the Isolink kit (Mallinckrodt Medical BV, Petten, The Netherlands) as previously described (44). Briefly, ^99m^Tc-tricarbonyl was complexed with the HIS_6_-tag at the C-terminal of the Nbs (50 µg) at 50°C. NAP-5 column (GE healthcare, Machelen, Belgium) followed by filtration through a 0.22 µm filter (Millipore, Haren, Belgium) was used to purify the ^99m^Tc-Nb complex. Labeling efficiency was evaluated with instant thin-layer chromatography (iTLC) before and after purification.

### Pinhole SPECT and micro-CT imaging and *ex vivo* biodistribution

Mice were injected with 5 µg ^99m^Tc-Nb with 54.686 ± 6.808 MBq one-hour before SPECT-CT imaging with the Vector+ scanner from MILabs B.V. (Houten, The Netherlands). The total body SPECT scan time was 20 min, 150 sec per position in spiral mode with six bed positions. The total CT time was 146 sec with parameters set to 60 kV and 615 mA. Images were analyzed with AMIDE (Medical Image Data Examiner software, UCLA, California, CA, USA) and OsiriX MD software (Bernex, Switzerland). Mice were sacrificed after imaging through cervical dislocation, organs of interest were collected and measured using the gamma counter (2480 WIZARD, PerkinElmer, Waltham, Massachusetts, US) to determine organ-specific uptake.

### Thermofluor assay

Fluorescent dye SYPRO Orange (5000X concentrated in DMSO, Life Technologies, Bleiswijk, The Netherlands) diluted with PBS to a final concentration of 1X was mixed with the Nbs (15 µM) to a final volume of 40 µL and qPCR machine (CFX connect™ Real-Time PCR system, Bio-Rad) was used to capture the dye signals at temperatures from 25-95°C with steps of 0.5°C.

### Statistical analyses

Graphpad Prism software was used to perform statistical analysis. One-way ANOVA, two-way ANOVA with multiple comparisons tests, or unpaired t-test were used to evaluate statistical significance. ns = p>0.05, * = p<0.05, ** = p<0.01, *** = p<0.001, **** = p<0.0001.

## Results

### Generation and characterization of Nbs against TIGIT

Llamas were immunized with recombinant m/h TIGIT recombinant proteins to generate affinity-matured heavy-chain only antibodies. Blood was collected from each llama and lymphocytes were isolated to create cDNA via PCR and cloning immune Nb libraries which were screened using phage display. After several rounds of panning on recombinant m/h TIGIT proteins and ELISA screenings, 154 different Nb sequences were identified, which could be divided into 43 families (B cell lineages) based on their CDR3.

Next, further screenings were performed using *E. coli* periplasmic extracts containing soluble Nbs. First, we evaluated the clones for binding to TIGIT expressed on cells. Therefore, HEK293T cells were stably transduced with lentiviral vectors encoding either m/h TIGIT, the untransduced HEK293T cells are referred to as wild type (WT) HEK293T cells. Successful TIGIT expression was validated by flow cytometry with fluorescent mAbs (**Supplementary Figure 2A**) and Nb binding was assessed (**Supplementary Figure 2B**). Additionally, the off-rates of the Nbs were determined using SPR on immobilized TIGIT proteins (**Supplementary Figure 3**). Based on the results generated from ELISA, flow cytometry cell binding study and off-rate screenings, nine Nbs binding to m/h TIGIT (indicated in **Table 1**, **Supplementary Figure 1**) were selected from five families for further characterization studies. Additionally, we designed and produced a scFv derived from the mAb Vibostolimab specific for hTIGIT (referred to as Vibo) and compared it side-by-side to the Nbs. The Nbs are species-specific, which is unsurprising based on the low homology (58%) between hTIGIT and mTIGIT (45). Throughout the study, an irrelevant Nb R3B23 binding to the 5T2 multiple myeloma (MM) produced M-protein was used as negative control (43).

**Table 1:** Summary of the characterization results of the Nbs. With the name of the Nb, production yields in *E. coli* per liter, K_D_ determined by SPR, and Tm.

mTIGIT Nbs	Yield (mg/L)	K_D_ (nM)	Tm (°C)
16966	5.4	0.157 ± 0.001	68.08 ± 0.22
16972	4.3	0.152 ± 0.001	69.63 ± 0.10
16979	0.75	845.867 ± 8.070	58.82 ± 0.75
16988	1.7	0.104 ± 0.001	71.98 ± 0.48

hTIGIT Nbs	Yield (mg/L)	K_D_ (nM)	Tm (°C)
16920	8.3	0.753 ± 0.002	63.15 ± 1.12
16925	3.8	1.974 ± 0.004	71.85 ± 0.55
17010	8.5	0.345 ± 0.001	65.87 ± 1.13
17018	15.1	0.470 ± 0.002	68.73 ± 1.33
17037	10.8	0.116 ± 0.001	59.30 ± 1.03
ScFv Vibo	9.7	0.996 ± 0.004	68.87 ± 0.33

K_D_, Equilibrium dissociation constant; Tm, Melting temperature; nM, nanomolar.

**Figure 1:**
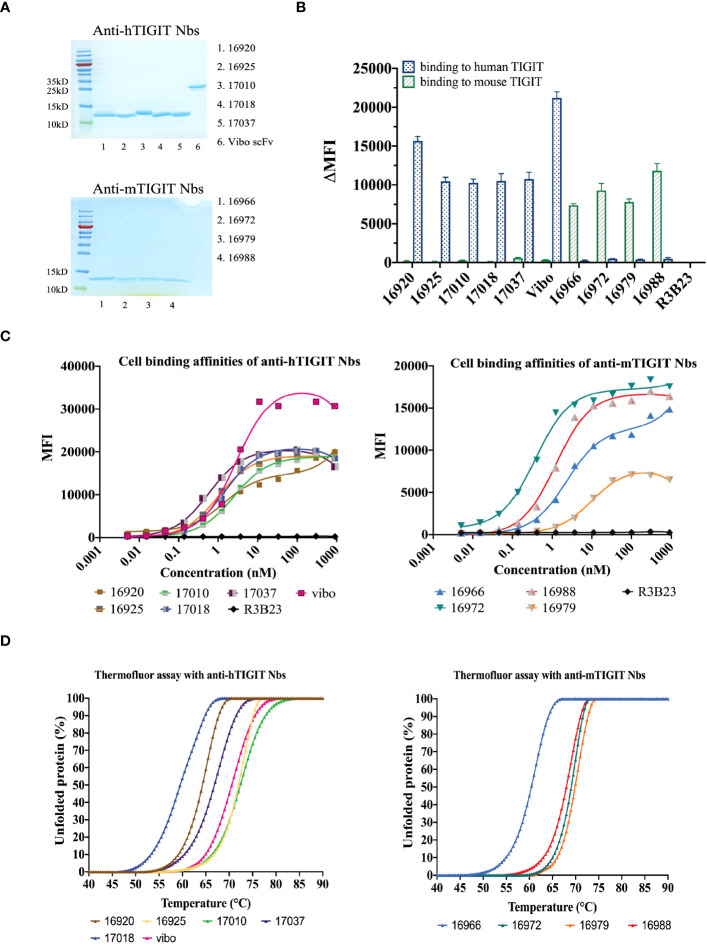
*In vitro* characterization of the purified Nbs. **(A)** SDS-PAGE of 5 µg purified Nbs or scFv Vibo stained with InstantBlue Coomassie protein stain. **(B)** Binding of 100 nM purified His_6_-tagged Nbs on mouse (black) or human (blue) TIGIT overexpressing HEK293T cells, detected with PE-labeled anti-His antibody and flow cytometry. Results are shown as delta mean fluorescent intensity (ΔMFI) by subtracting the MFI of Nb binding on WT HEK293T cells from Nb binding on m/h TIGIT expressing HEK293T cells. **(C)** Affinity of the purified Nbs on HEK293T cells transduced to express human (left panel) or mouse TIGIT (right panel), detected by flow cytometry using anti-His antibody and a dilution range of Nbs. **(D)** Percentage unfolded Nbs or scFv Vibo at different temperatures, determined by the Thermofluor assay.

The selected Nbs and scFv were produced as HIS_6_-tagged proteins with yields between 0.75 and 15.1 mg/L and purified with SEC (**Table 1**). SDS-PAGE confirmed the purity of the produced Nbs and scFv and detected the expected molecular weight following Instant Blue Coomassie staining (**Figure 1A**). The Nbs and Vibo scFv binding affinities were evaluated using SPR on immobilized recombinant TIGIT proteins. Most of the Nbs and Vibo scFv demonstrated a fast association and a slow dissociation, which resulted in sub-nanomolar affinities for m/h TIGIT (**Table 1**, **Supplementary Figure 4**, **Supplementary Table 1**). Using flow cytometry, the Nbs and Vibo scFv bound to either mTIGIT^+^ or hTIGIT^+^ HEK293T cells but not to WT HEK293T cells (**Figure 1B**). The good affinities were also confirmed on cells expressing m/h TIGIT (**Figure 1C**). Only Nb 16979 showed a sub-optimal affinity towards mTIGIT with a fast dissociation and was subsequently excluded from further experiments. Finally, the Nbs and the Vibo scFv thermal stability was determined using a Thermofluor assay. The melting temperature (Tm) of Nbs is a crucial parameter since radiolabeling with ^99m^Tc requires temperatures up to 50°C making Nbs with a higher Tm more favorable. All the Nbs and the scFv Vibo showed a Tm above 50°C, with Nb 16925 (anti-hTIGIT) and Nb 16988 (anti-mTIGIT) showing the highest Tm (71.85 ± 0.55°C and 71.98 ± 0.48°C respectively) (**Table 1**, **Figure 1D**).

**Figure 2:**
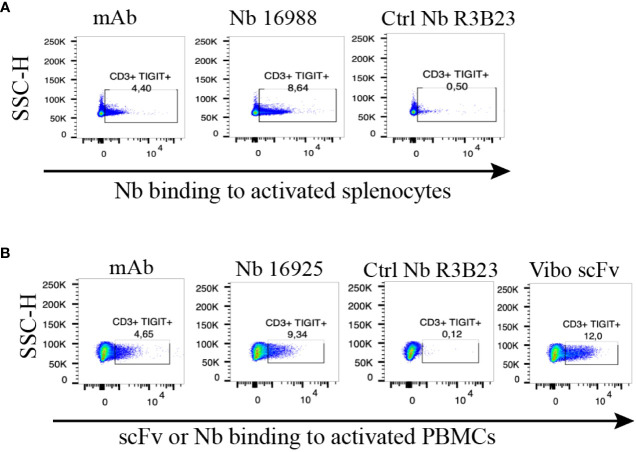
Anti-TIGIT Nbs bind to TIGIT expressed on splenocytes or PBMCs, results from the lead Nbs are shown. **(A)** anti-mTIGIT Nb 16988 incubated with 900 nM to CD3/28 dynabeads-activated mouse splenocytes, compared to the irrelevant ctrl Nb R3B23 and the commercially available mAb. **(B)** anti-hTIGIT Nb 16925 or Vibo ScFv incubated with 900 nM to CD3/28 dynabeads-activated human PBMCs, compared to the irrelevant ctrl Nb R3B23 and the commercially available mAb. Binding of Nb or scFv was detected with PE-labeled anti-His antibody on flow cytometry.

To ensure the produced Nbs and Vibo scFv could recognize and bind to physiologically expressed TIGIT, we assessed their binding on mouse splenocytes or human PBMCs stimulated with anti-CD3/CD28 dynabeads. Flow cytometry confirmed TIGIT expression on different subsets of immune cells, including CD3^+^, CD4^+^ and CD8^+^ T cells, and Nb binding was detected on these cells (**Figure 2**, **Supplementary Figure 5**). The Vibo scFv showed higher binding percentage compared to the Nbs.

### Anti-TIGIT Nbs accumulate specifically in tumors with overexpressed TIGIT

To assess whether the anti-TIGIT Nbs and the Vibo scFv accumulate specifically in tumors with high TIGIT expression, *in vivo* SPECT-CT imaging with ^99m^Tc-labeled Nbs and Vibo scFv was performed. Immunodeficient mice were subcutaneously inoculated in the flanks with both untransduced TC-1 lung carcinoma cells (referred to as WT TC-1) and TC-1 cells overexpressing either mouse or human TIGIT. Of note, the transduced TC-1 cells express more TIGIT (74.7% with ΔMFI of 3031 for the mTIGIT transduced TC-1) (**Supplementary Figure 6**) compared to the physiological situation (with a ΔMFI of 24.6 on 4.40% of the CD3^+^ splenocytes). The expression of TIGIT did not influence tumor growth (**Supplementary Figure 6**). After radiolabeling with ^99m^Tc and purification, all Nbs and scFv showed >98% radiochemical purity (RCP), assessed with iTLC (**Supplementary Figure 7**). Tumor bearing mice were injected with 5µg of radiolabeled Nbs with an activity of 54.686 ± 6.808 MBq. SPECT-CT imaging was performed one hour post-injection, followed by an *ex vivo* biodistribution study including gamma-counting of dissected organs to determine tracer uptake in tissues, shown as percentage injected activity per gram (%IA/g). Uptake of the radiolabeled Nbs is expected in the kidneys and bladder due to renal clearance of the tracer, specific signal is expected in the m/h TIGIT overexpressing tumors but not in the TIGIT negative tumor nor with a radiolabeled irrelevant Nb.

**Figure 3:**
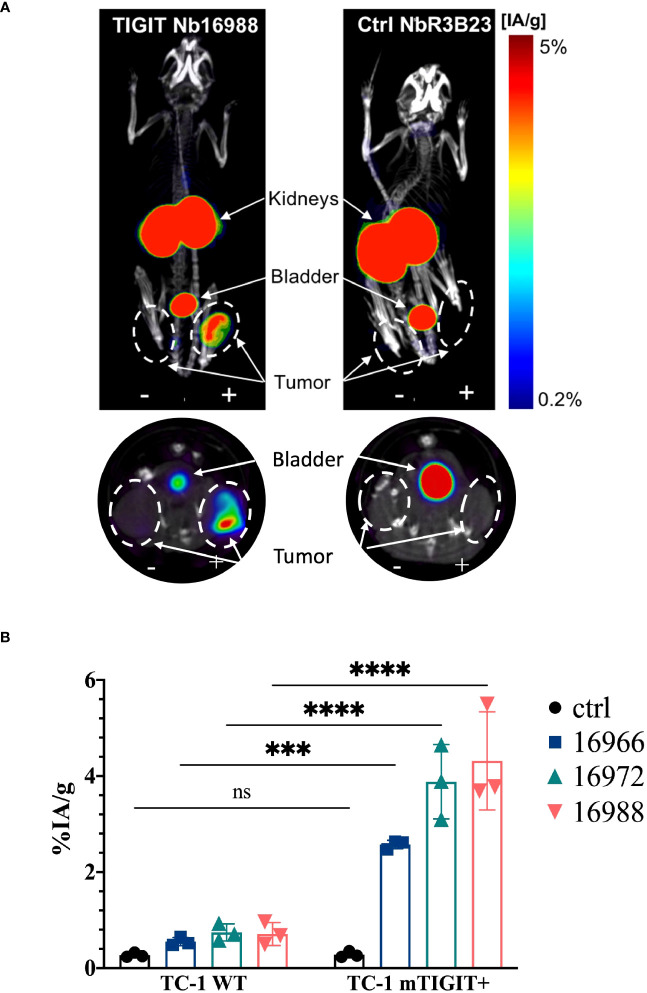
*In vivo* SPECT-CT imaging and *ex vivo* biodistribution of the anti-mTIGIT Nbs in immunodeficient mice bearing TC-1 tumors (n=3). **(A)** 3D-rendered SPECT-CT images (top) and a transversal slice at the level of the tumors (bottom) of a representative mouse bearing a mTIGIT-transduced (+) and an untransduced (-) TC-1 tumor and injected with ^99m^Tc-labeled anti-mTIGIT Nb 16988 (left) or control Nb R3B23 (right). **(B)** *ex vivo* biodistribution results of the control Nb and the three selected anti-mTIGIT Nbs showing percentage injected activity per gram (%IA/g) tissue in WT TC-1 and mTIGIT^+^ TC-1 tumors. Two-way ANOVA was used to calculate statistical significance. Statistical significance was set at p<0.05 (ns, not significant, ***=p<0.001, ****=p<0.0001).

**Figure 4:**
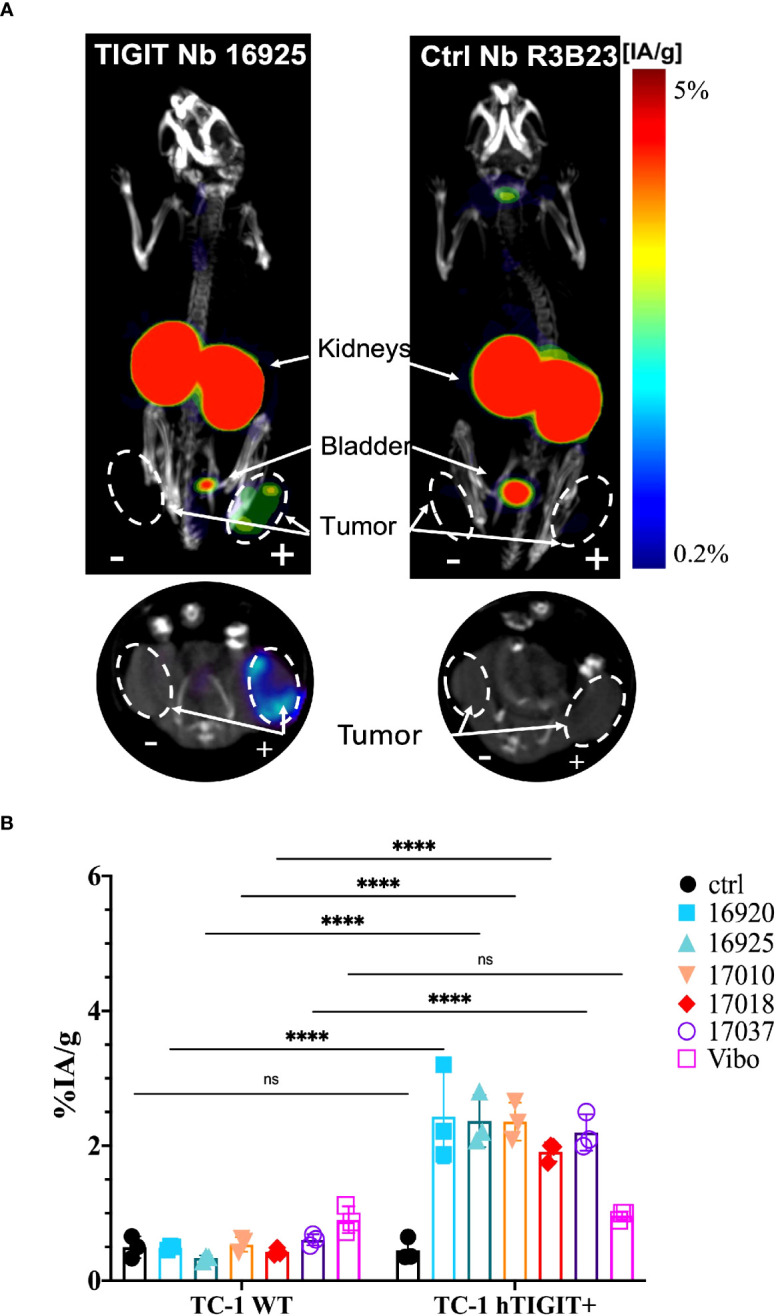
*In vivo* SPECT-CT imaging and *ex vivo* biodistribution of the anti-hTIGIT Nbs in immunodeficient mice bearing TC-1 tumors (n=3). **(A)** 3D-rendered SPECT-CT images (top) and a transversal slice at the level of the tumors (bottom) of a representative mouse bearing a hTIGIT-transduced (+) and an untransduced (-) TC-1 tumor and injected with ^99m^Tc-labeled anti-hTIGIT Nb 16925 (left) or control Nb R3B23 (right). **(B)** *ex vivo* biodistribution results of the control Nb and the selected anti-hTIGIT Nbs showing percentage injected activity per gram (%IA/g) tissue in WT TC-1 and hTIGIT^+^ TC-1 tumors. Two-way ANOVA was used to calculate statistical significance. Statistical significance was set at p<0.05 (ns, not significant, ****=p<0.0001).

High uptake of the anti-TIGIT tracers in the kidneys, bladder, and the m/h TIGIT overexpressing tumors could be seen on the SPECT-CT images (**Figures 3A**, **4A**, **Supplementary Figures 8A**, **9A**). The control Nb R3B23 did not show any signal within the tumors and the Vibo scFv showed signals in both WT and hTIGIT-overexpressing tumors. The *ex vivo* biodistribution confirmed the findings of the SPECT-CT images. A typical biodistribution profile of the Nbs was obtained, with high kidney retention due to renal clearance, fast blood clearance and overall low uptake in normal tissues (**Supplementary Figures 8B**, **9B**). In TIGIT-overexpressing TC-1 tumors, up to 4.317 ± 1.021%IA/g in mTIGIT TC-1 (**Figure 3B**) and 2.431 ± 0.692%IA/g in hTIGIT TC-1 tumors (**Figure 4B**) could be detected with respective anti-m/hTIGIT Nbs compared to 0.282 ± 0.065%IA/g with the irrelevant control Nb. The uptake of the anti-m/hTIGIT Nb tracers was not significantly different compared to the uptake of the irrelevant control Nb in WT TC-1 tumor. The Vibo scFv could not discriminate between WT and hTIGIT-overexpressing tumors at one hour post-injection (**Figure 4B**) and its uptake in the tumors is comparable to that of the normal tissues (**Supplementary Figure 9B**). Despite the higher expression of hTIGIT compared to mTIGIT on TC-1 cells, the anti-hTIGIT Nb 16925 exhibited nearly two-fold less uptake within the tumor compared to anti-mTIGIT Nb 16988. This observation may indicate that the anti-hTIGIT Nb is relatively less effective than the anti-mTIGIT Nb.

**Figure 5:**
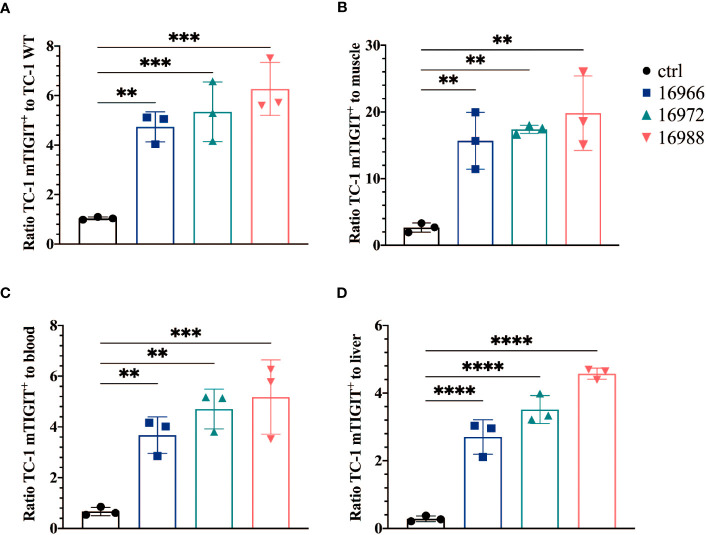
Anti-mTIGIT Nbs show high signal-to-noise ratios compared to the control Nb R3B23. Ratios of uptake of ^99m^Tc-labeled anti-mTIGIT Nbs in TC-1 mTIGIT^+^ tumor to **(A)** TC-1 WT tumor, **(B)** muscle, **(C)** blood or to **(D)** liver (n=3), ratios are calculated as following: uptake in TC-1 mTIGIT^+^ divided by uptake in TC-1 WT tumor, muscle, liver, or blood. One-way ANOVA was used to evaluate statistical significance. Statistical significance was set at p<0.05 (**=p<0.01, ***=p<0.001, ****=p<0.0001).

**Figure 6:**
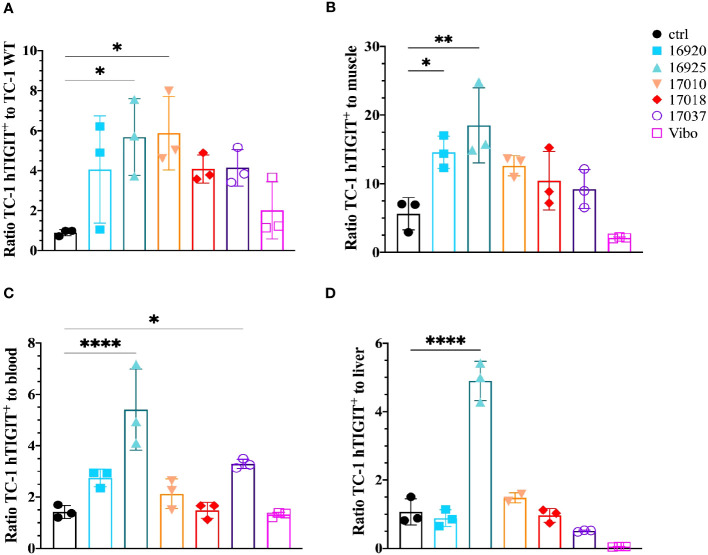
Anti-hTIGIT Nbs show high signal-to-noise ratios compared to the control Nb R3B23 and the scFv Vibo. Ratios of uptake of ^99m^Tc-labeled anti-hTIGIT Nbs in TC-1 hTIGIT^+^ tumor to **(A)** TC-1 WT tumor, **(B)** muscle, **(C)** blood or to **(D)** liver (n=3), ratios are calculated as following: uptake in TC-1 hTIGIT^+^ divided by uptake in TC-1 WT tumor, muscle, liver, or blood. One-way ANOVA was used to evaluate statistical significance. Statistical significance was set at p<0.05 and only shown for significant data (*=p<0.05, **=p<0.01, ****=p<0.0001).

We also evaluated the tumor-to-background ratios of the Nbs and compared this to the irrelevant control Nb. The anti-hTIGIT Nb 16925 and anti-mTIGIT Nb 16988 showed the highest tumor-to-background ratios in all conditions and were chosen as our lead Nbs (**Figures 5**, **6**).

**Figure 7:**
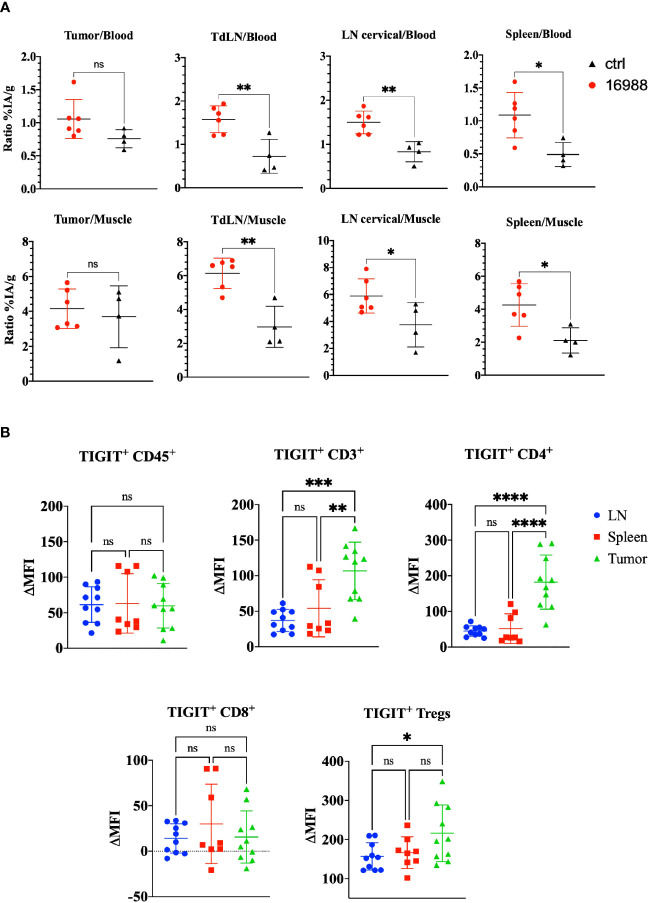
*Ex vivo* biodistribution of ^99m^Tc-labeled anti-mTIGIT Nb 16988 in C57BL/6 mice bearing a subcutaneous MC38 tumor. **(A)** Ratio %IA/g of tumor, tumor draining lymph node (TdLN), cervical LN, spleen to blood or to muscle. **(B)**. mTIGIT expression (ΔMFI) evaluated on single cells suspensions of the lymph node, spleen, and MC38 tumor on CD45^+^, CD3^+^, CD4^+^, CD8^+^ and CD25^+^ CD127^-^ Tregs using flow cytometry by subtracting the MFI of the fluorescence minus one (FMO) from the signal. Unparied t-test **(A)** or one-way ANOVA **(B)** was used to evaluate statistical significance. Statistical significance was set at p<0.05 (ns, not significant, *=p<0.05, **=p<0.01, ***=p<0.001, ****=p<0.0001).

Having demonstrated the specific targeting and high uptake of the mTIGIT Nb tracer 16988 in an mTIGIT overexpressing tumor model *in vivo*, our objective was to assess the potential of Nb 16988 in imaging mTIGIT expression on tumor-infiltrating lymphocytes (TILs) in tumor-bearing immunocompetent mice. Therefore, MC38 CRC cells were inoculated subcutaneously in the flank of C57BL/6 mice, a model that was shown before to contain TIGIT^+^ TILs (46). At day 14 post-inoculation, we conducted *in vivo* SPECT-CT imaging using ^99m^Tc-labeled Nb 16988 or the irrelevant control Nb, and subsequently evaluated tissue uptake levels *ex vivo*. However, no detectable tumor uptake was observed on the SPECT-CT images. We compared the signal-to-noise ratios of Nb 16988 to that of the control Nb (**Figure 7A**). Although a higher tumor-to-blood ratio was observed with Nb 16988, this difference was not statistically significant compared to the control Nb. Nevertheless, the lymph node or spleen-to-blood or -to-muscle ratios of Nb 16988 were significantly higher than those of the control Nb. Moreover, we performed flow cytometry analysis to evaluate TIGIT expression ex vivo in tumor, spleen, or lymph node as single cell suspensions, focusing on the CD45^+^, CD3^+^, CD4^+^, CD8^+^ and Treg populations (**Figure 7B**). The expression of TIGIT was found to be low and highly variable within the group of mice (n=10). No significant difference in TIGIT expression could be detected between the lymph node, spleen, and tumor from the CD45^+^ TILs. These results highlight the correlation between the low expression of TIGIT on TILs and the limited tumor uptake *in vivo* with ^99m^Tc labeled Nb 16988.

### SPECT-CT imaging with ^99m^Tc labelled anti-TIGIT Nbs in human TIGIT KI mice demonstrate specificity *in vivo*

**Figure 8:**
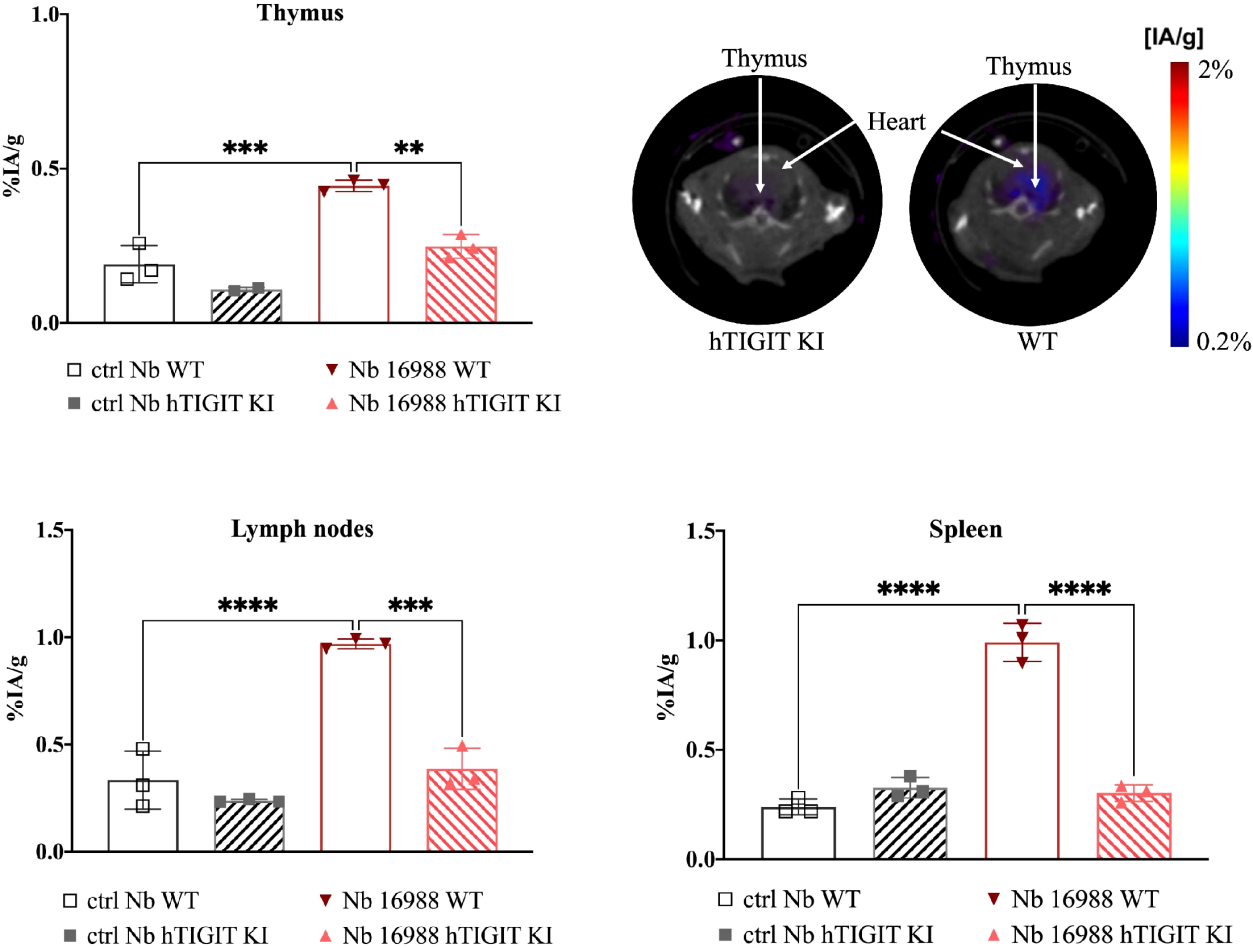
*In vivo* SPECT-CT imaging and ex vivo biodistribution of ^99m^Tc-radiolabeled anti-mTIGIT Nb 16988 and irrelevant control Nb R3B23 in wild type C57BL/6 mice and hTIGIT KI mice. Biodistribution study with ^99m^Tc-Nb16988 showing uptake (%IA/g) in the thymus, spleen and lymph nodes with SPECT-CT imaging analyzed with Amide showing a transversal slice of the thymus uptake in hTIGIT KI mouse compared to WT mouse. Statistical analyses were performed using one-way ANOVA. Statistical significance was set at p<0.05 (**=p<0.01, ***=p<0.001, ****=p<0.0001).

**Figure 9:**
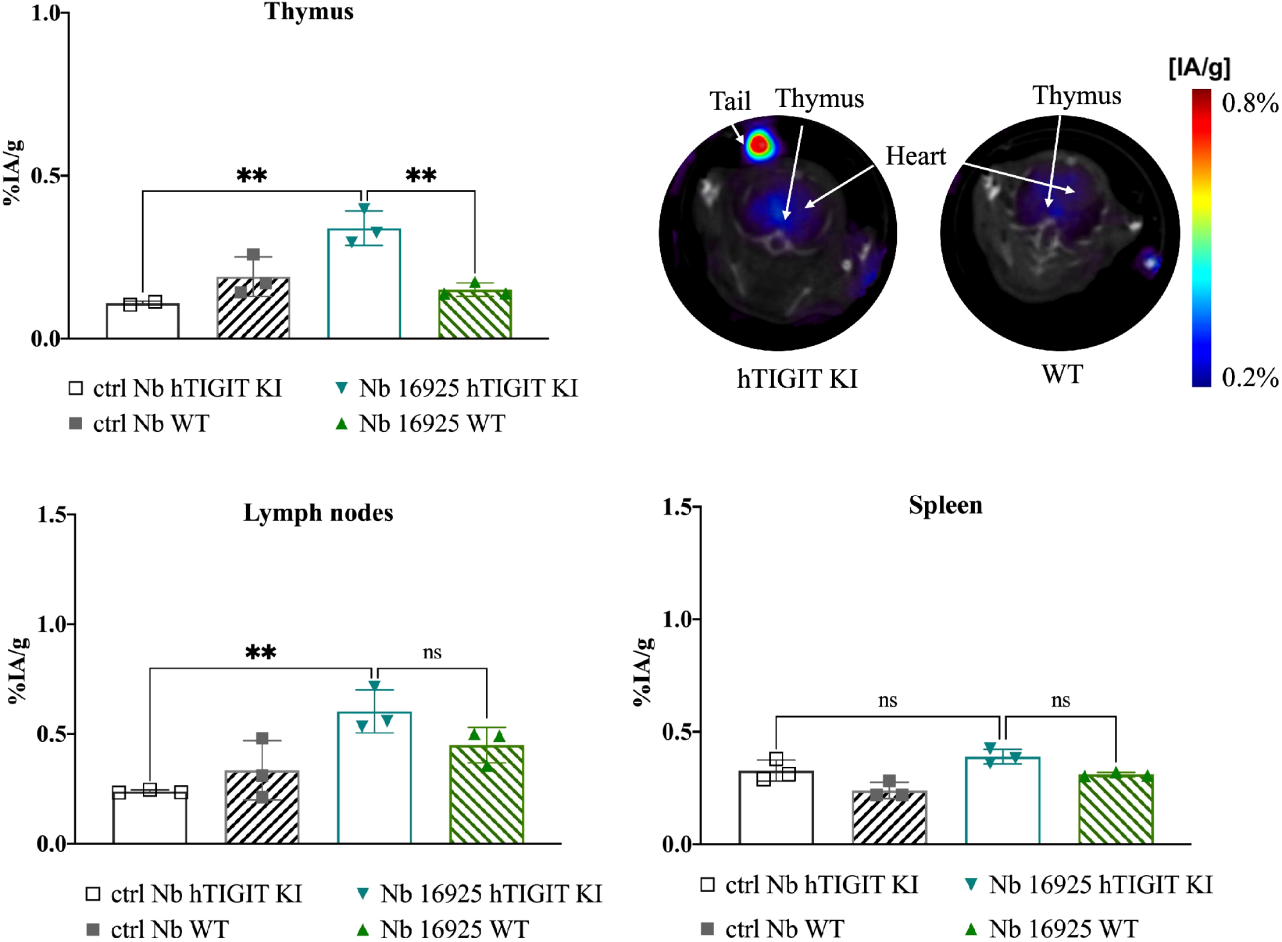
*In vivo* SPECT-CT imaging and *ex vivo* biodistribution of ^99m^Tc-radiolabeled anti-hTIGIT Nb 16925 and irrelevant control Nb R3B23 in wild type C57BL/6 mice and hTIGIT KI mice. Biodistribution study with ^99m^Tc-Nb16925 showing uptake (%IA/g) in the thymus, spleen and lymph nodes with SPECT-CT imaging analyzed with Amide showing a transversal slice of the thymus uptake in hTIGIT KI mouse compared to WT mouse. Statistical analyses were performed using one-way ANOVA. Statistical significance was set at p<0.05 (ns, not significant, **=p<0.01). .

To further validate the specificity of the lead mTIGIT and hTIGIT Nb tracers, we performed SPECT-CT imaging and *ex vivo* biodistribution analyses comparing WT C57BL/6 mice with hTIGIT KI mice, where the extracellular domain of mTIGIT was replaced with hTIGIT (Shanghai Model Organisms Center, Inc.). In this way the radiolabeled anti-mTIGIT Nb should have lower uptake compared to the naïve C57BL/6 mice, and higher uptake with radiolabeled anti-hTIGIT Nb. As expected, the irrelevant control Nb showed no significant difference in organ uptake between WT and hTIGIT KI mice. In contrast, the ^99m^Tc-labeled anti-mTIGIT Nb 16988 exhibited significantly higher uptake in the spleen, lymph nodes, and thymus of WT C57BL/6 mice when compared to the hTIGIT KI mice (**Figure 8**). This elevated uptake in the thymus was also evident on the SPECT-CT imaging (**Figure 8**). As for the hTIGIT Nb 16925, a significant higher uptake in the thymus was detected in hTIGIT KI mice compared to the WT mice, this could also be captured on the SPECT-CT imaging (**Figure 9**). However, no significantly higher uptake in the lymph node and spleen in hTIGIT KI mice compared to WT mice was observed. These data provide additional confirmation of the specificity of the lead Nb 16988 and Nb 16925 as imaging tracers for mTIGIT and hTIGIT, respectively. Nonetheless, it is important to note that the hTIGIT targeting tracer demonstrated lower performance than the mTIGIT Nb 16988 *in vivo.*

## Discussion

TIGIT is a next-generation ICP and increasing numbers of mAbs targeting TIGIT have entered clinical trials, such as Tiragolumab (Genentech), Vibostolimab (Merck) and Etigilimab (Mereo BioPharma). However, the clinical outcome of these mAbs has not been uniformly positive. For instance, while the CITYSCAPE II trials with Tiragolumab (Genentech) showed promising results in patients with lung carcinoma, disappointing outcomes were observed in two phase III trials (SKYSCRAPER-01, SKYSCRAPER-02) involving patients with SCLC and NSCLC (27). Only a fraction of patients responded positively to anti-TIGIT therapy. As a result, there is a critical need for noninvasive methods to quantify and monitor TIGIT expression, which could potentially assist in patient stratification and improve the response to therapy.

In this study, we described the generation, production, and characterization of a subset of Nbs specific for the ICP TIGIT as radiolabeled tracer to noninvasively image TIGIT. 154 different Nbs from 43 different CDR families were screened. Four Nbs were found binding to mTIGIT, and five Nbs binding to hTIGIT. Binding was evaluated on cell lines transduced with m/h TIGIT but also on physiologically expressed mTIGIT on splenocytes and hTIGIT on PBMCs. Low nanomolar binding affinities of the mTIGIT lead Nb 16988 and the hTIGIT lead Nb 16925 were determined by SPR and on cells transduced to express or naturally expressing TIGIT.

To the best of our knowledge, this is the first study evaluating anti-TIGIT Nbs as tracers for noninvasive imaging. *In vivo* SPECT-CT studies with the anti-mTIGIT tracers in naïve mice showed overall low signal, but tracer uptake was detected in the lymph nodes, spleen, thymus. The specificity of lead anti-mTIGIT Nb tracer was further confirmed *in vivo* in hTIGIT KI mice that do not express mTIGIT. Compared to the uptake in the naïve mice, a significant lower accumulation was detected in all organs described above. In mTIGIT-overexpressing tumor bearing mice, the lead anti-mTIGIT Nb tracer 16988 showed specific binding to mTIGIT within the tumor and generated high tumor-to-background ratios already after one hour post-injection. Despite observing some live uptake, the tumor-to-liver ratio is higher compared to that of the control Nb. To comprehensively assess the potential of the Nbs to noninvasively quantify TIGIT^+^ TILs, we performed imaging and quantification of TIGIT in immunocompetent mice bearing a syngeneic tumor. Specifically, the MC38 tumor model was used which has been previously reported to contain TIGIT^+^ TILs (46) and was validated through flow cytometry analysis (**Figure 7B**). We detected 9.48 ± 3.36% CD45^+^ cells within the MC38 tumor microenvironment. Among the CD45^+^ population, 8.39 ± 4.20% cells exhibited TIGIT expression, and this proportion was further identified as 4.46 ± 3.71% within the CD4^+^ population. Notably, this percentage is much lower compared to what has been described by Chen et al. with 13.6% TIGIT^+^ CD4^+^ cells. As such, the low tumor uptake (0.629 ± 0.092%IA/g) of the Nb tracer 16988 is most likely correlated with the low levels of TIGIT expression on TILs within the MC38 tumor in our study. Kurtulus et al. showed TIGIT expression within the tumor microenvironment of the melanoma B16F10 tumor model (12) and other tumor models such as CT26 (47), EMT6 breast carcinoma (48), GL261 glioblastoma (49), A20 lymphoma (50) have been used to study the effect of anti-TIGIT-blocking mAbs. It would be worthwhile to also investigate the potential of our Nbs to detect TIGIT in these models.

In recent years, other TIGIT tracers have been reported. Shaffer et al. described a mAb-based mTIGIT specific tracer radiolabeled with Copper-64 (^64^Cu) or Zirconium-89 (^89^Zr). Their study demonstrated uptake in xenografts and syngeneic mouse tumor models after 48 or 72 hours of injection. However, due to the long circulation time and slow blood clearance of these tracers, achieving high-contrast imaging within a short time was challenging. This was evidenced by 29.3 + 4.5%ID/g tumor uptake and blood activity of 9.7 + 1.0%ID/g at 72 hours with the ^89^Zr labeled tracer (51). In contrast, the lead anti-mTIGIT Nb tracer described in this study showed specific uptake with 4.317 ± 1.0121%ID/g as early as one hour after injection, with low blood activity (0.936 ± 0.545%ID/g) in TC-1 tumor bearing mice.

Another study conducted by Weng et al. reported a peptide-based Galium-68 (^68^Ga) PET radiotracer to evaluate TIGIT expression in mice bearing 4T1 breast cancer. The affinity of this peptide is notably higher (4.1 µM) in comparison to the here reported Nb tracers, which exhibited affinities within the sub-nanomolar range. Despite this, tumor uptake (1.1 ± 0.19%ID/g) was detectably with the peptide radiotracer as early as 0.5 h post-injection. However, *ex vivo* analysis revealed significant liver and blood uptake (52).

The above-described studies further supported the promising diagnostic potential of small antigen-binding moieties, such as Nbs, for the noninvasive detection and quantification of targets within tumors within a short timeframe. Consequently, the use of these moieties may help to reduced the radiation burden associated with imaging procedures.

In this study, we compared the ability of radiolabelled Nbs to a mAb-derived scFv Vibo to detect TIGIT expression at one hour post-injection using nuclear imaging *in vivo*. While the *in vitro* characteristicsc of the scFv Vibo is comparable to the Nbs, including affinity and thermal stability, its *in vivo* performance was sub-optimal. This was demonstrated by the inability of the scFv Vibo to discriminate tumors with high TIGIT expression from tumors with low TIGIT expression (**Figures 4**, **6**). Moreover, a much higher accumulation of the scFv Vibo in the liver, spleen and several other organs is detected compared to the Nbs (**Supplementary Figure 8**).

A potential rationale for this observation is that in general scFv’s can suffer from instability and aggregation issues due to the configuration of the heavy and light chain variable domains (53). More specifically, the hydrophobic interface from framework 2 to facilitate VH and VL joining lowers the solubility of the scFv, resulting in higher aggregation potential (54). Protein aggregation is known to induce liver and spleen sequestration through macrophage phagocytosis, which might explain the high liver uptake of the scFv Vibo and aspecific signals demonstrated by *in vivo* imaging studies. Furthermore, the scFv’s molecular weight, being twice that of a Nb, might result in less efficient tissue penetration capacity. This has been suggested by Debie et al. who showed that bivalent Nbs as compared to their monovalent form accumulate slower at target site, which is most likely due to their doubled size (55). On the contrary, the small size of the Nbs allows them to diffuse faster within tissues and enhanced accessibility to bind on hidden or cryptic epitopes of the antigens (54). A comprehensive and more in depth investigation will be needed to unravel the underlying mechanisms responsible for the *in vivo* behavior of the scFv Vibo.

Previously, Nbs targeting immune checkpoints LAG-3 and PD-L1 have been developed by Lecocq et al. (34) and Broos et al. (36), allowing fast and high-contrast imaging upon radiolabeling. In the MC38 model, Lecocq et al. showed 1.2%IA/g uptake in MC38 tumor and this percentage increased to 2.1%IA/g when treated with PD-1 antibody. The expression of LAG-3 within CD45^+^ cells is around 10% and when treated with PD-1 increased to approximately 16% with the MFI between 200-600, whilst TIGIT only showed a MFI of 95.4 ± 3.2 within the CD45^+^ population. Considering that TIGIT expression is much lower than the expression of LAG-3 or PD-L1 within the tumor, it seems to be challenging to quantify TIGIT expression using nuclear imaging in tumor models with low TIGIT expression, like the MC38 tumor model used in this study. In the B16 syngeneic tumor model Shaffer et al. described above, 8.88% to 12.39% TIGIT-positive cells were detected within the live cells from the tumor microenvironment, while a tumor uptake of 7.4 + 0.9%ID/g in the nonblocking condition was shown (51). Further studies with other tumor models with higher TIGIT expression are needed to fully evaluate the diagnostic potential of the anti-mTIGIT Nb tracer described in this study.

The hTIGIT-targeting Nbs also showed promising results in mice bearing hTIGIT-overexpressing tumors. High-contrast imaging was possible at one hour post tracer administration. Lead hTIGIT Nb 16925 demonstrated specific accumulation in tumors with high hTIGIT expression. The tumor-to-background ratios were higher compared to the control Nb. A ^68^Ga-labeled D-peptide antagonist was reported by Wang et al. for PET imaging of TIGIT expression and evaluated the safety and potential for TIGIT imaging in two patients with advanced NSCLC, which is so far the only clinically-evaluated hTIGIT-targeting imaging probe (56).

To conclude, the Nb tracers showed promising potential to detect TIGIT noninvasively with high tumor-to-background ratios one hour post-injection with optimal tracer characteristics such as low-sub-nanomolar binding affinities and high thermal stability. The low tumor uptake in the syngeneic tumor model is possibly due to the low TIGIT expression within the tumor. Other tumor models will be evaluated for TIGIT expression and Nb tracer uptake upon nuclear imaging. Combination therapy with other therapies to enhance TIGIT expression will be needed to fully validate the Nb tracers. Nevertheless, the obtained results showed promising diagnostic potential of Nbs to noninvasively image high TIGIT expression within the tumor, and hold promise for clinical translation to aid patient selection and improve therapy response.

## Data availability statement

The datasets presented in this study can be found in online repositories. The names of the repository/repositories and accession number(s) can be found in the article/**Supplementary Material**.

## Ethics statement

The animal study was approved by Ethical Committee for Use of Laboratory Animals of the VUB. The study was conducted in accordance with the local legislation and institutional requirements.

## Author contributions

KZ: Conceptualization, Methodology, Writing – original draft, Writing – review & editing, Data curation, Formal Analysis, Funding acquisition. TD: Data curation, Writing – review & editing. HC: Data curation, Writing – review & editing. RA: Writing – review & editing. TE: Writing – review & editing. Wd: Writing – review & editing. FM: Writing – review & editing. GR: Supervision, Writing – review & editing. KB: Conceptualization, Supervision, Writing – review & editing. ND: Conceptualization, Funding acquisition, Project administration, Supervision, Writing – review & editing.
