# Supplementary material for: Development and evaluation of nanobody tracers for noninvasive nuclear imaging of the immune-checkpoint TIGIT
Source: Front Immunol. 2023 Sep 20;14:1268900. doi: 10.3389/fimmu.2023.1268900 (PMC10548220; doi:10.3389/fimmu.2023.1268900)
Supplement: Supplementary file 1 [file DataSheet_1.docx]

Supplementary Material

# Supplementary Figures and Tables

## Supplementary Figures


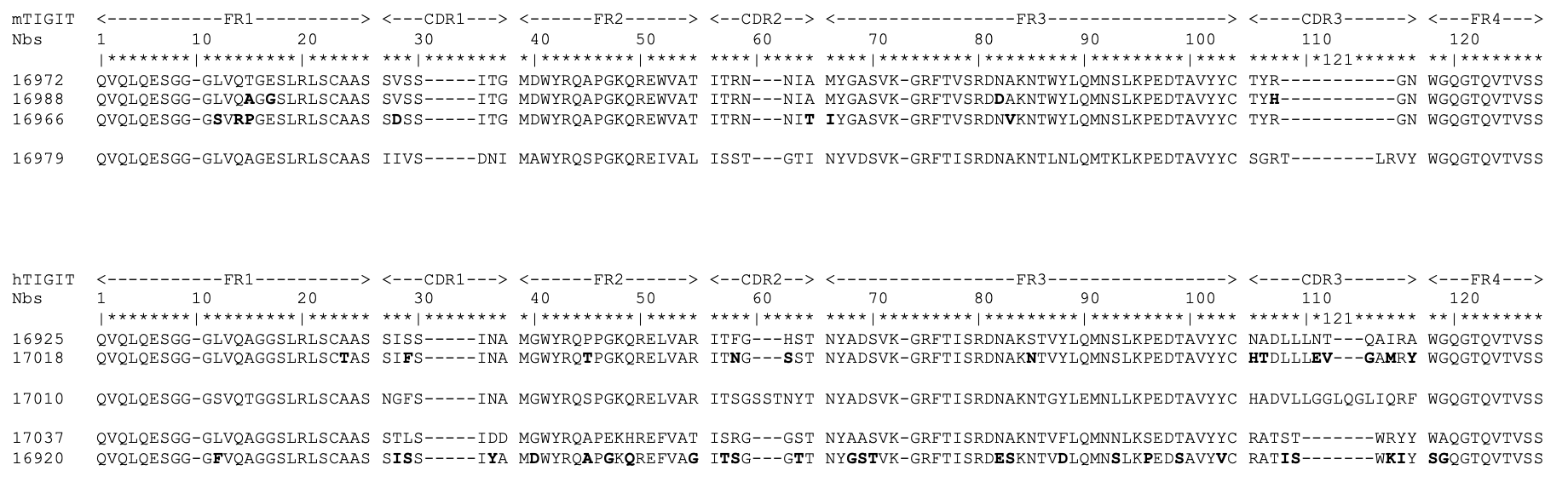
 Supplementary Figure 1. Amino acid sequences of the nine selected Nbs shown according to IMGT numbering and grouped according to sequence similarity.

**
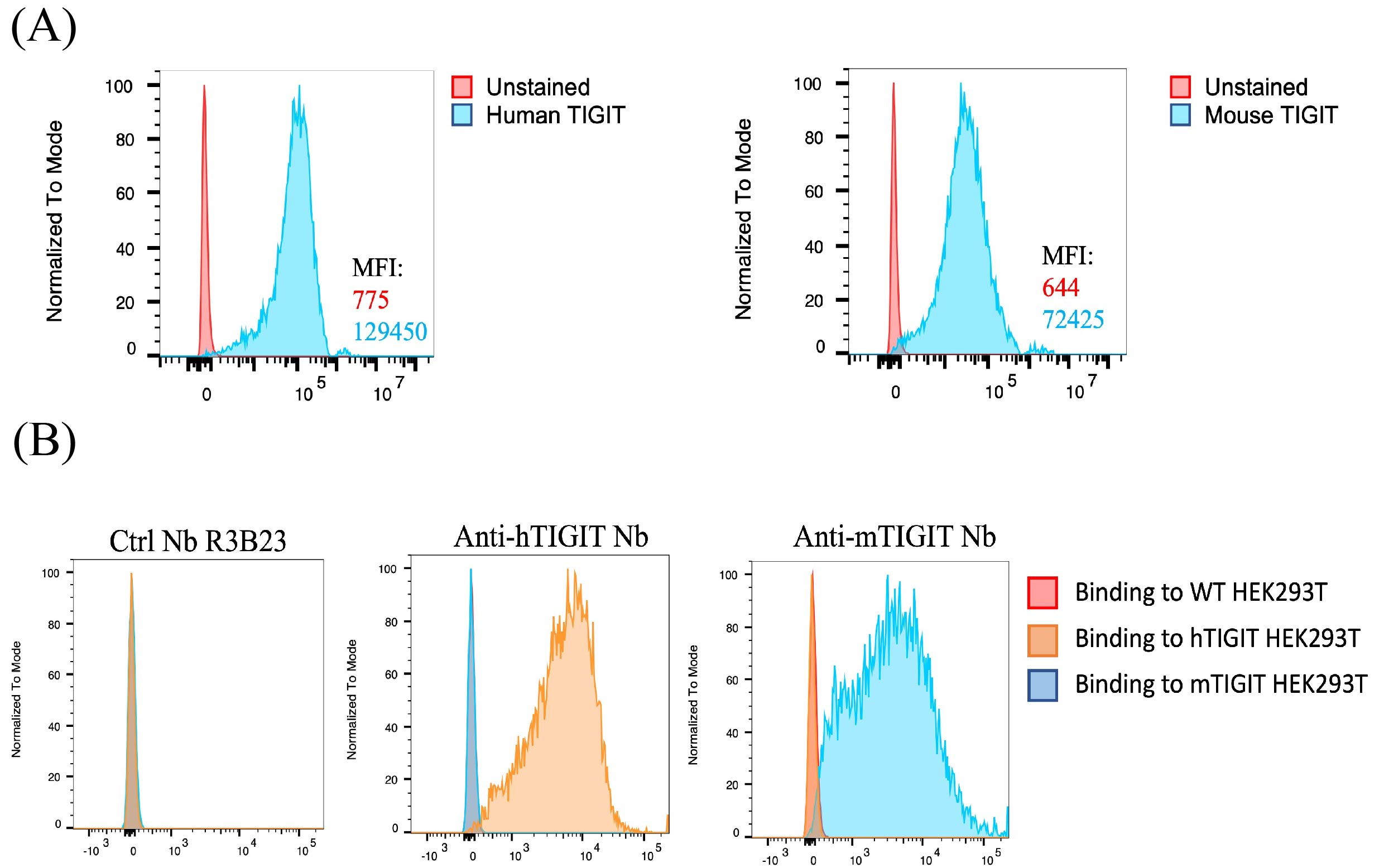
**

**Supplementary Figure 2.** **Transduction of HEK293T cells and binding of Nbs within the periplasmic extracts to untransduced (WT), h/m TIGIT+ HEK293T cells detected by flow cytometry.** (A) HEK293T cells transduced with lentiviral vectors to express mTIGIT or hTIGIT, transduction was successful and stable, as evaluated with flow cytometry using a PE-labeled antibody specific for mTIGIT or hTIGIT (blue), compared with the unstained cells (red). (B) Example of a Nb show binding to hTIGIT^+^ HEK293T cells (middle) and a Nb show binding to mTIGIT^+^ HEK293T cells (Right). The control Nb R3B23 show no binding to untransduced or transduced HEK293T cells (Left). Nb binding was detected with flow cytometry using APC-labelled anti-HA antibody.


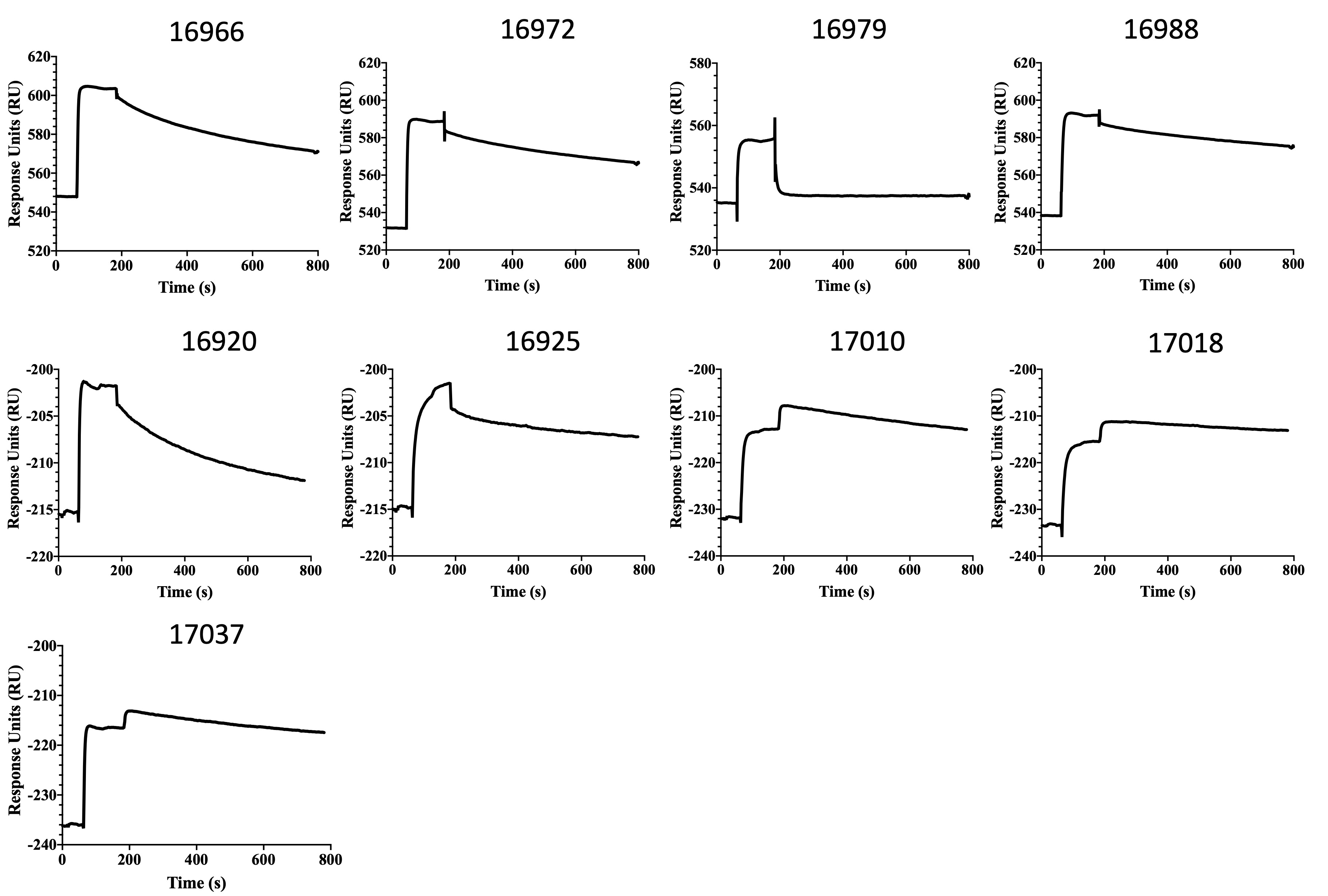


**Supplementary Figure 3. Off-rate determination using surface plasmon resonance (SPR) with Biacore T200.** The graphs showing the response units (RU) over 800 seconds (s) of the periplasmic extracts containing TIGIT binding Nbs on mouse or human TIGIT recombinant protein coated CM5 chip.


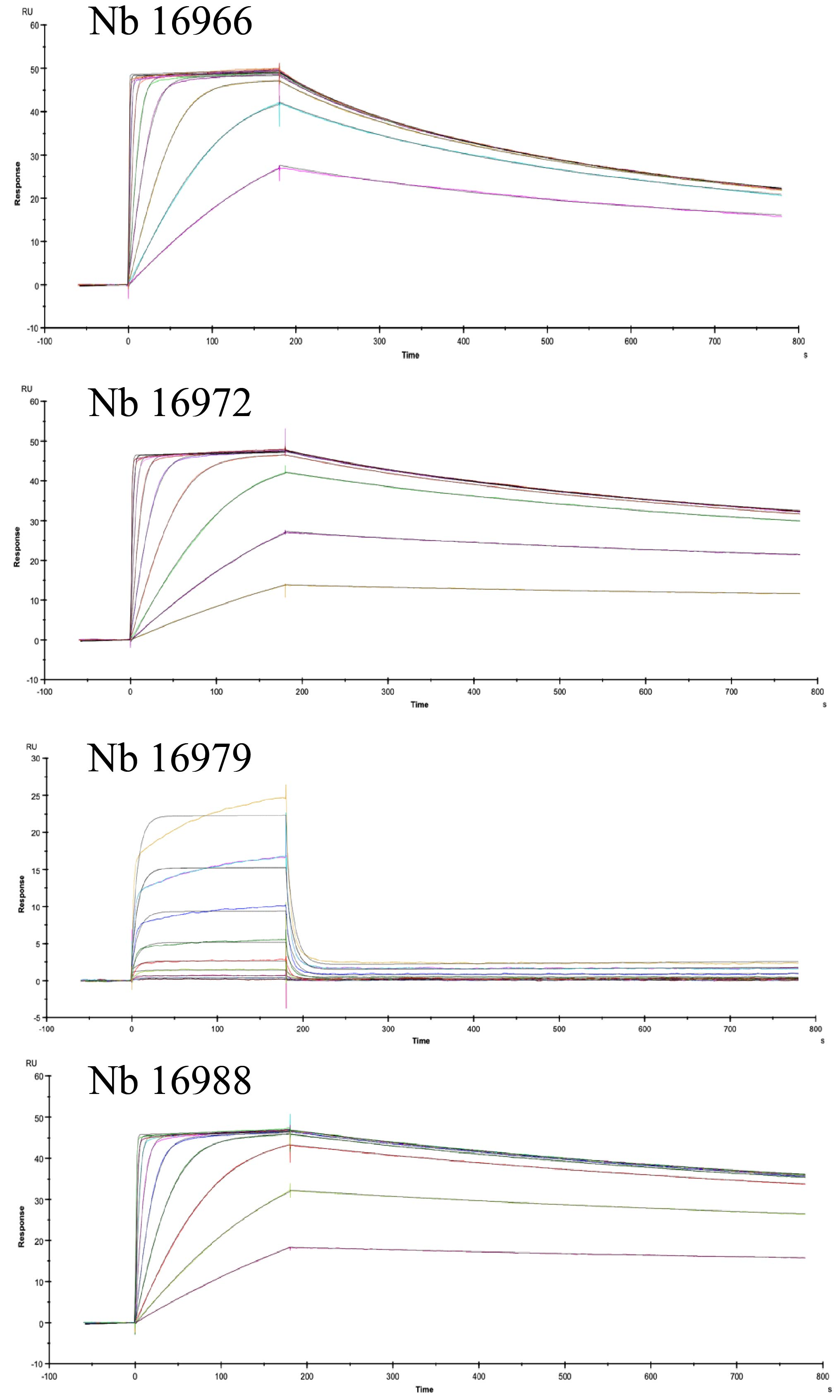


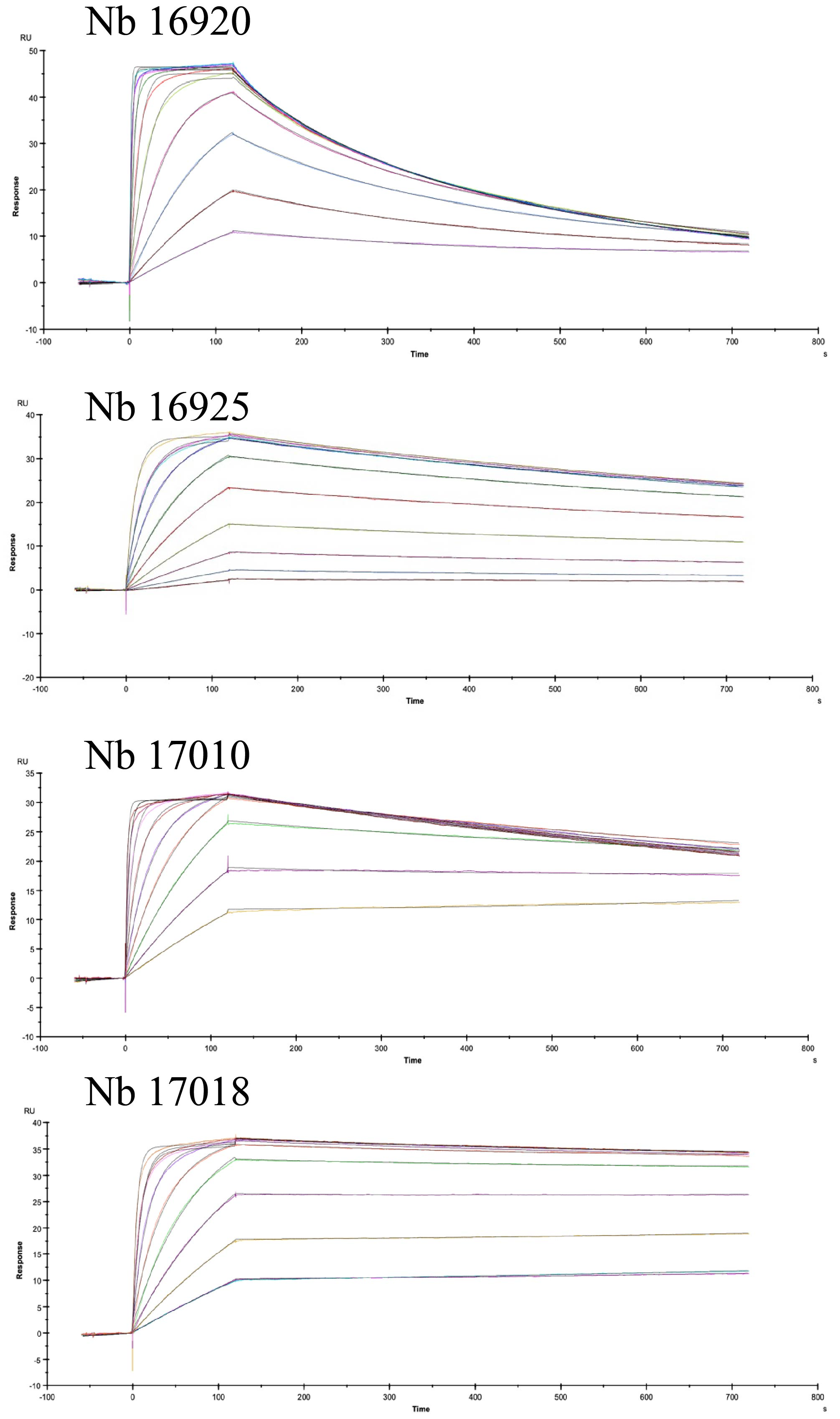


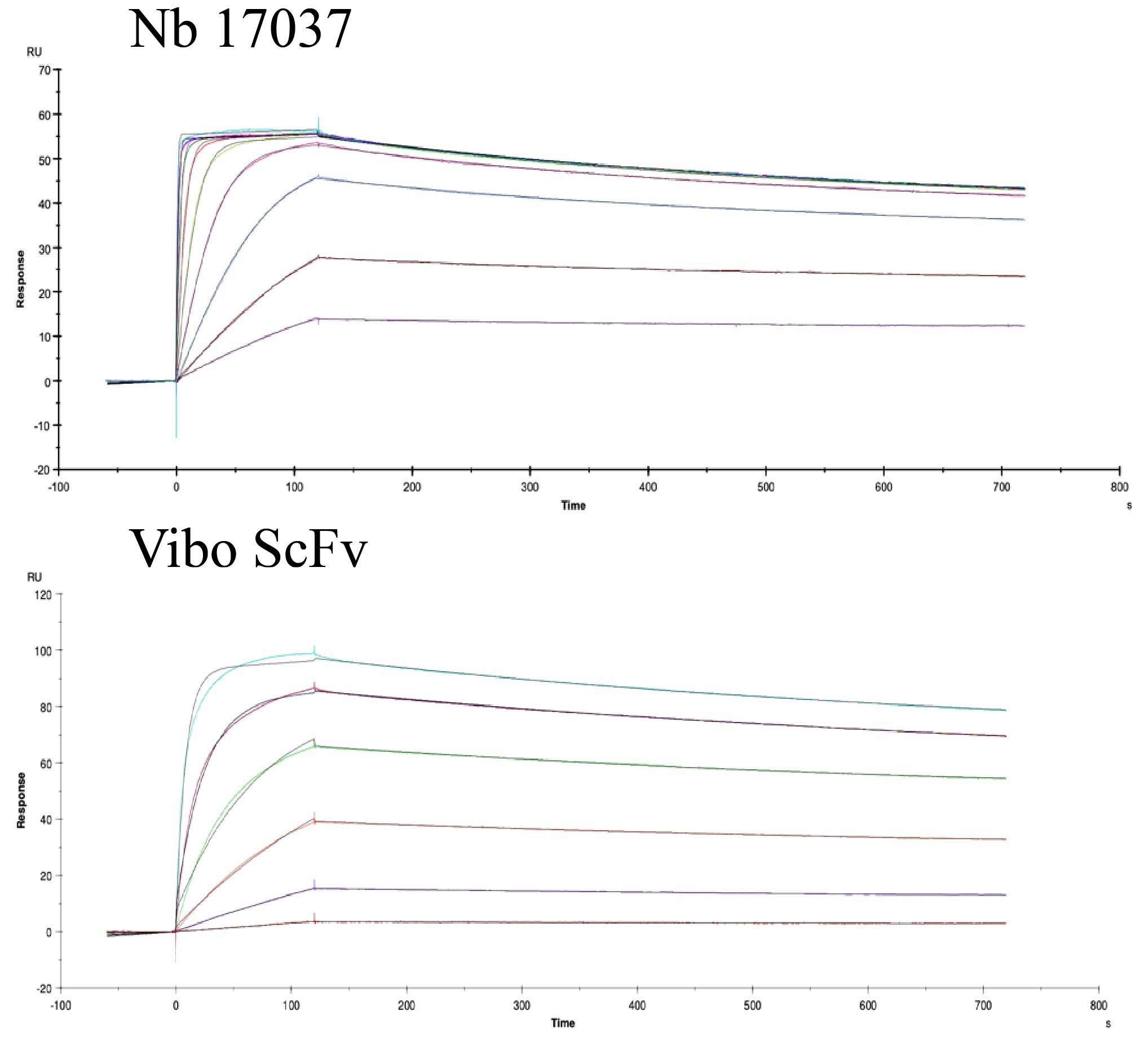


**Supplementary Figure 4. Affinity/kinetics of the anti-m/hTIGIT Nbs determined by SPR on the Biacore T200.** The fitting of the binding curves using the 1:1 binding with drift and RI2 model was applied to calculate kinetic parameters. The following concentration of the Nbs or scFv were evaluated: 200nM, 100nM, 100nM, 50nM, 25nM, 12.5nM, 6.25nM, 3.125nM, 1.56nM, 0.78nM.


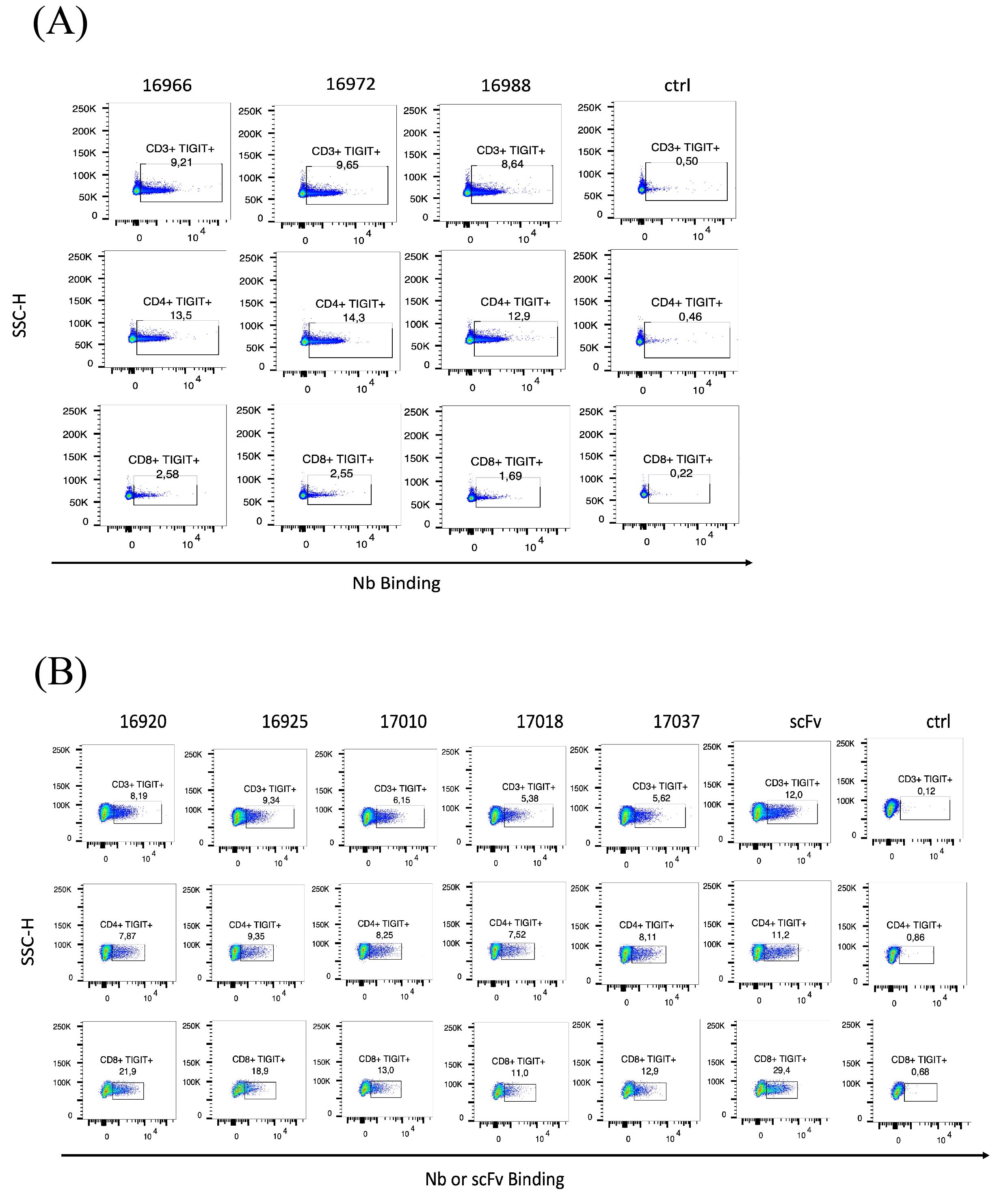


**Supplementary Figure 5**. **Anti-TIGIT Nbs bind to TIGIT expressed on splenocytes or PBMCs**. (A) anti-mTIGIT Nbs at a concentration of 900 nM incubated with CD3/28 dynabeads-activated mouse splenocytes, Nb binding was detected using flow cytometry with PE-labelled anti-His antibody. (B) anti-hTIGIT Nbs or scFv at a concentration of 900 nM incubated with CD3/28 dynabeads-activated PBMCs. Nb binding was detected using flow cytometry with PE-labelled anti-His antibody.


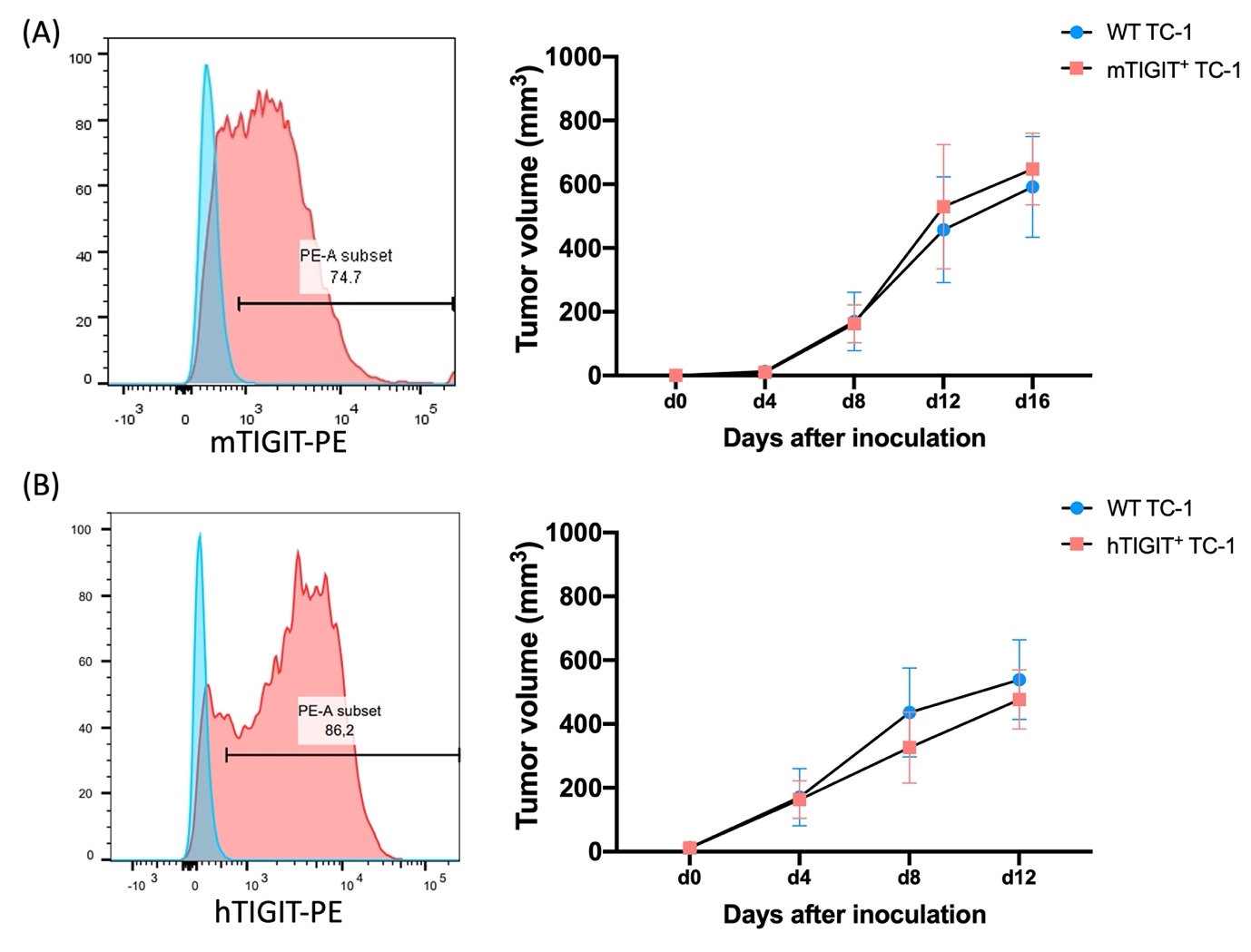


**Supplementary Figure 6. Transduction of TC-1 cells to overexpress m/h TIGIT evaluated with flow cytometry and the tumor growth curves of untransduced and m/h TIGIT^+^ TC-1 cells *in vivo*.** (A) Left: TC-1 cells transduced with lentiviral vectors encoding mTIGIT (red) evaluated with flow cytometry using PE labeled anti-mTIGIT antibody, compared to mTIGIT expression on WT TC-1 cells (blue). Right: Tumor growth of the subcutaneously implanted WT TC-1 (blue) and mTIGIT TC-1 (red) cells in immunodeficient mice (n=12). (B) Left: TC-1 cells transduced with lentiviral vectors encoding hTIGIT (red) evaluated with flow cytometry using PE labeled anti-hTIGIT antibody, compared to hTIGIT expression on WT TC-1 cells (blue). Right: Tumor growth of the subcutaneously implanted WT TC-1 (blue) and hTIGIT TC-1 (red) cells in immunodeficient mice (n=21).


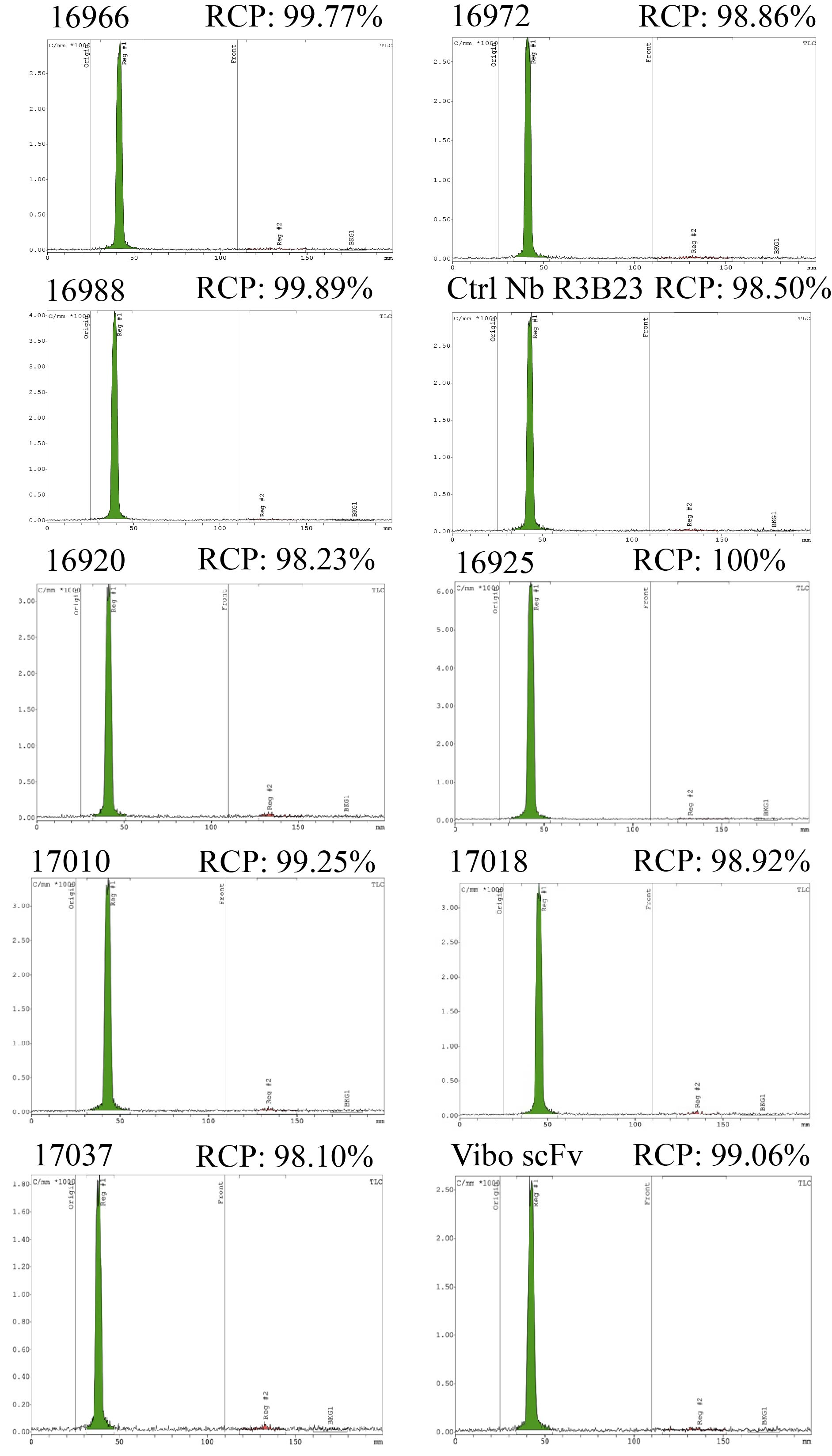


**Supplementary Figure 7. iTLC of the Nbs and scFv Vibo after ^99m^Tc labeling**, evaluated with the Elysia Raytest and data analysis was done by Gina Star TLC software, showing >98% RCP.


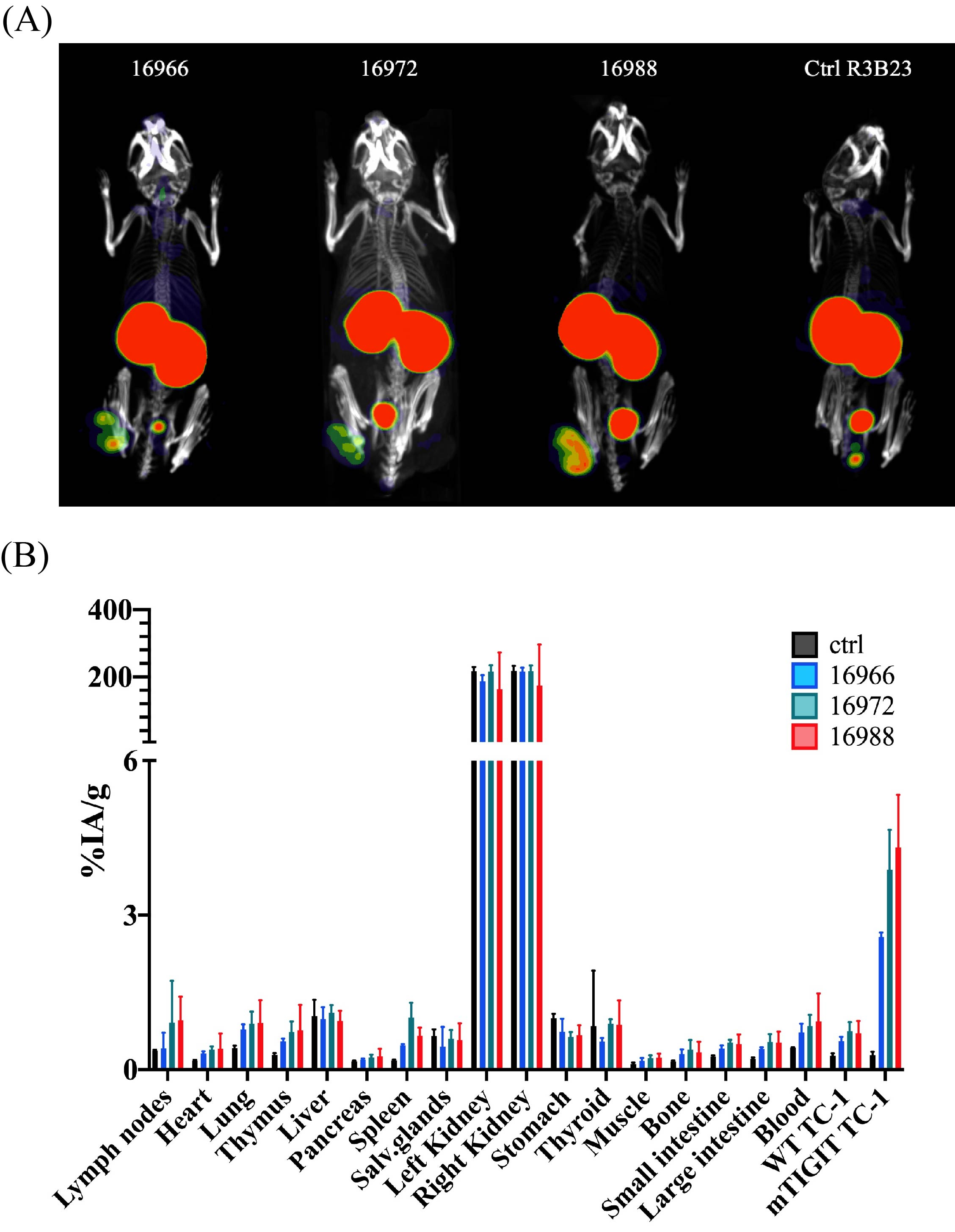


**Supplementary Figure 8. *In vivo* SPECT-CT imaging and *ex vivo* biodistribution of ^99m^Tc-labeled anti-mTIGIT Nbs in immunodeficient mice bearing mTIGIT overexpressing TC-1 and WT TC-1 tumors (n=3).** (A) 3D-rendered SPECT/CT images of a representative mouse bearing a mTIGIT-transduced (+) and an untransduced (-) TC-1 tumor and injected with ^99m^Tc-labeled anti-mTIGIT Nbs or the control Nb R3B23. (B) *ex vivo* biodistribution results of the control Nb and the selected anti-mTIGIT Nbs showing percentage injected activity per gram (%IA/g) organ/tissue indicated on the graph.


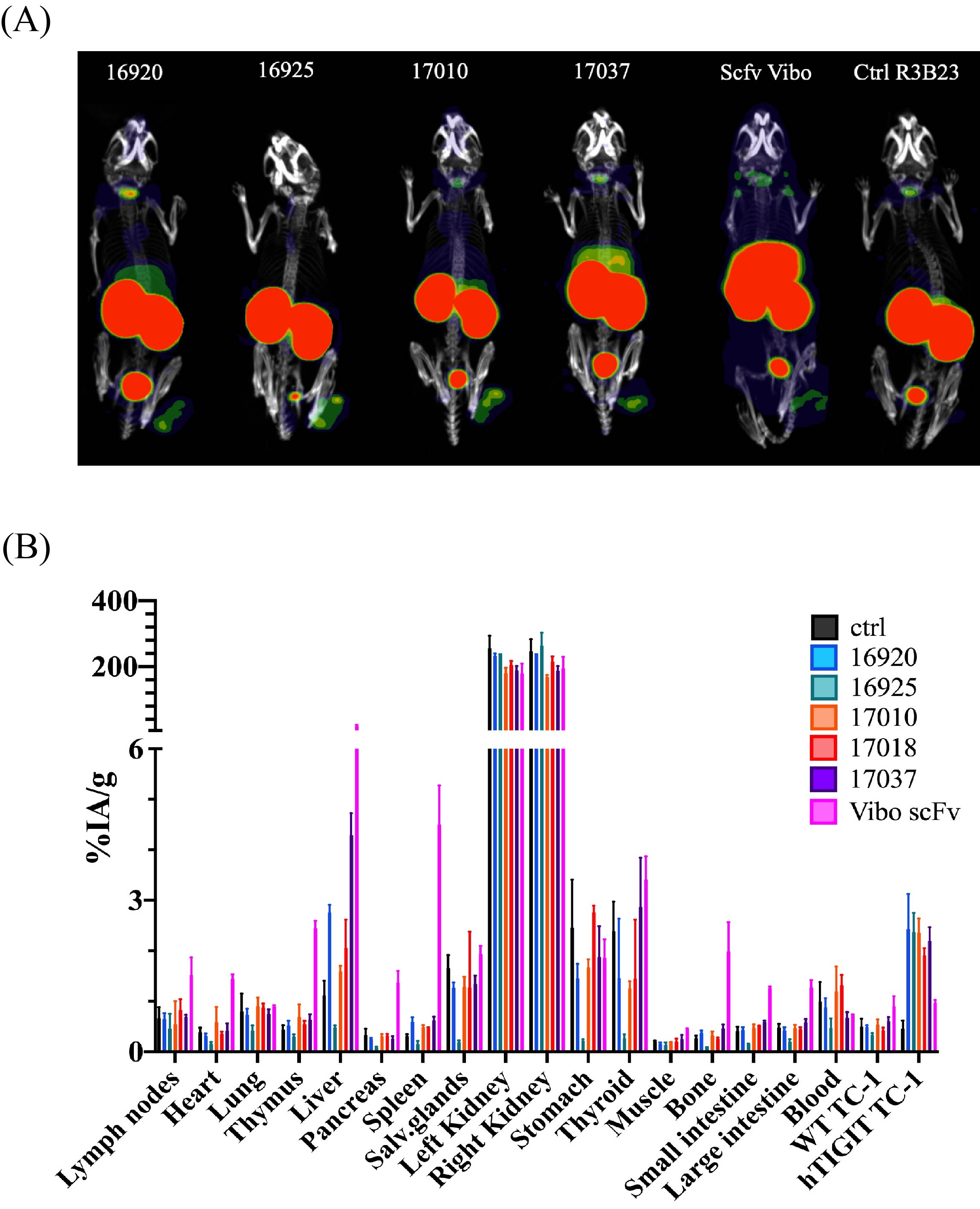


**Supplementary Figure 9. *In vivo* SPECT-CT imaging and *ex vivo* biodistribution of ^99m^Tc-labeled anti-hTIGIT Nbs in immunodeficient mice bearing hTIGIT overexpressing TC-1 and WT TC-1 tumors (n=3).** (A) 3D-rendered SPECT/CT images of a representative mouse bearing a hTIGIT-transduced (+) and an untransduced (-) TC-1 tumor and injected with ^99m^Tc-labeled anti-hTIGIT Nbs, scFv or the control Nb R3B23. (B) *ex vivo* biodistribution results of the control Nb and the selected anti-hTIGIT Nbs or scFv showing percentage injected activity per gram (%IA/g) organ/tissue indicated on the graph.

**Supplementary Table 1. Affinity/kinetics of the anti-m/hTIGIT Nbs determined by SPR on the Biacore T200.** Showing the k_a_, k_d_ and K_D_ of the Nbs.

| Anti-mTIGIT Nb | k_a_ (1/Ms) | k_d_ (1/s) | K_D_ (nM) |
| --- | --- | --- | --- |
| 16966 | 1.640E+07 | 2.571E-03 | 0.157 |
| 16972 | 7.462E+06 | 1.133E-03 | 0.152 |
| 16979 | 1.343E+05 | 1.136E-01 | 845.867 |
| 16988 | 7.064E+06 | 7.376E-04 | 0.104 |
| Anti-hTIGIT Nb | k_a_ (1/Ms) | k_d_ (1/s) | K_D_ (nM) |
| 16920 | 5.663E+06 | 4.262E-03 | 0.753 |
| 16925 | 4.616E+05 | 9.113E-04 | 1.974 |
| 17010 | 2.326E+06 | 8.026E-04 | 0.345 |
| 17018 | 1.056E+06 | 4.965E-04 | 0.470 |
| 17037 | 1.362E+07 | 1.573 E-03 | 0.116 |
| ScFv Vibo | 8.296E+05 | 8.268E-04 | 0.996 |

k_a_: Association constant; k_d_: Dissociation constant; K_D_: Equilibrium dissociation constant

**Supplementary Table 2. Summary of antibodies used for surface cell stainings for flow cytometry analysis.**

| Antibodies  (for *ex vivo* single cell suspensions) | Fluorophore | Clone |
| --- | --- | --- |
| CD45.2 | VioBlue | REA1223 |
| CD3 | FITC | REA641 |
| CD4 | VioBright-R720 | REA604 |
| CD8a | APC-Vio770 | REA601 |
| CD25 | PE-Vio770 | REA568 |
| CD127 | APC | REA680 |
| CD19 | PerCP-Vio770 | REA749 |
| NK1.1 | APC | REA1162 |
| TIGIT | PE | REA536 |

| Antibodies  (for PBMC Nb binding) | Fluorophore | Clone (Cat.No.) |
| --- | --- | --- |
| CD3 | APC | HIT3a (Biolegend, 300312) |
| CD4 | PerCP-Cy5.5 | L200 (BD Biosiences, 552838) |
| CD8 | BV510 | SK1 (BD Biosciences, 563919) |
| CD25 | Alexa Fluor 488 | M-A251 (Biolegend, 356116) |
| CD127 | APC-eFluor 780 | eBioRDR5  (ThermoFisher Sienctific, 47-1278-42) |
| CD56 | PE-Cy7 | 5.1H11 (Biolegend, 362510) |
| TIGIT | PE | A15153G (Biolegend 372704) |

| Antibodies  (for splenocyte Nb binding) | Fluorophore | Clone (Cat.No.) |
| --- | --- | --- |
| CD45.2 | APC-Cy7 | 104 (BD Biosciences, 560694) |
| CD3 | PE-Cy7 | 17A2 (Biolegend, 100220) |
| CD19 | PerCP-Cy5.5 | 1D3 (BD Biosciences, 551001) |
| CD4 | Alexa Fluor 700 | RM4-5 (BD Biosciences, 557956) |
| CD8a | V450 | 53-6.7 (BD Biosciences, 560471) |
| TIGIT | PE | A17200C (Biolegend, 622206) |
